# Impact of digital health interventions for adolescents with type 1 diabetes mellitus on health literacy: a systematic review

**DOI:** 10.1186/s12902-023-01321-6

**Published:** 2023-03-31

**Authors:** Aurélia Naoko Naef, Christoph Wilhelm, Hürrem Tezcan-Güntekin, Volker Eric Amelung

**Affiliations:** 1 Institute for Epidemiology, Social Medicine and Health Systems Research, Hannover Medical School, Hannover, Germany; 2 Harding Center for Risk Literacy, Faculty of Health Sciences, University of Potsdam, Potsdam, Germany; 3 Department of Health and Education, Alice Salomon Hochschule Berlin, Berlin, Germany; 4 Berlin School of Public Health, Charité Universitätsmedizin Berlin, Berlin, Germany

**Keywords:** Type one diabetes mellitus, Adolescents, Digital health interventions, Health literacy, Compliance

## Abstract

**Background:**

Evidence shows that living with diabetes mellitus type 1 (T1DM) in adolescent age is particularly challenging and difficult to manage. A high level of health literacy is important to prevent and avoid debilitating complications. Despite the increasing prevalence and incidence of T1DM by adolescent and the large use of digital health interventions, little is known about the association between this use and health literacy. This systematic review provides an overview on the impact of digital health interventions for adolescents with type 1 diabetes on health literacy and derive recommendations for further research.

**Methods:**

Electronic searches were performed in five databases in Medline (Medline, PubMed + via PubMed), The Cochrane Library, EMBASE (via Ovid), Web of Science and PsycINFO from 2011 to 2021. In addition, grey literature searches were conducted in Google Scholar, OAlster and Trip. Relevant studies that have been missed by electronic and hand-searching strategies were searched in the reference lists of all included studies. The review followed PRISMA guidelines. Two researchers independently screened abstracts for initial eligibility and applied the inclusion and exclusion criteria to the relevant full-text articles. Quality was assessed using the tools RoB2 Cochrane, ROBINS I, NOS (Newcastle–Ottawa Scale), CASP (Critical Appraisal Skills Programme) for primary studies and Amstar-2 for secondary studies.

**Results:**

Out of 981 studies, 22 were included in the final review. Most primary studies included in this review were judged as moderate overall risk of bias or with some concerns and most of the secondary studies as critically low quality reviews. Our findings suggest that the interplay of health care providers (HCP) and patients through social media helps the management of the disease. This corroborates Bröder et al.’ (2017) dimension of ‘communication and interactions’ in their concept of health literacy.

**Conclusions:**

For adolescents with T1DM, social media may be a specific and beneficial intervention for an improved communication and interaction with their HCP. Further research should investigate what specific form of social media suits best for which adolescents.

**Trial registration:**

The study protocol was registered on the 15th of November 2021 on Prospero (reg. NR: CRD42021282199).

**Supplementary Information:**

The online version contains supplementary material available at 10.1186/s12902-023-01321-6.

## Background

According to the International Diabetes Federation [1], more than 1.2 million children and adolescents (0–19 years) are living with diabetes mellitus type 1 (T1DM) worldwide in 2021. This number increases by approximately 3% annually [2]. The chronic disease, which is described by the World Health Organisation (WHO) as a ‘global epidemic’, has taken on unprecedented importance in the world's healthcare system [3]. The same issues exist on a national level: the prevalence and incidence of T1DM are also increasing in Germany. According to the Diabetes’ German Health Report, more than 32,500 children and young people under the age of 20 are affected. 3,100 new cases of T1DM are estimated to occur in Germany each year [4]. The management of this disease is particularly challenging for adolescents, who are already in a vulnerable developmental stage of life [5]. In general, patients have to integrate a lot of information and combine them with practical skills and competences [6]. They have to adhere to an intensive and complex daily regimen, such as the monitoring of blood glucose level, the estimating nutritional intake, the dosing of insulin multiple times per day. Furthermore, there are psychosocial issues like stigma, stress, burn-out, peer relation and diabetes-related family conflicts [7]. For Bakhach and colleagues [8], this ‘diabetes distress’ consists of feelings of frustration, hopelessness, anger, guilt or fear. Hence, the importance of a high level of health literacy, especially for young people with chronic diseases, is no longer questioned by the scientific community.

Improving health literacy through digital tools could help to get a more direct contact to adolescents also outside the clinic and practice, so that these daily issues may be addressed even better in the future. Indeed, the rapid advancement of digital tools has contributed to the transformation of health care in the last decade, is also part of the daily life of adolescents and could be integrated as a support to manage their chronic diseases. According to the JIM Study 2020 [9], 89% of young people are online every day with an average of 4.3 h, which provides a great potential for digital tools in adolescents with common chronic diseases such T1DM. Improving diabetes self-management skills via promoting health literacy through an age-appropriate strategy and with digital tools could be the key in order to prevent complications, may increase their quality of life and have a significant impact on clinical outcome [8, 10, 11, 7]. Nevertheless, the evidence is inconsistent [12].

This study aims to provide an overview of the literature on the current evidence regarding the impact of digital health interventions (distal technologies according to the definition of Duke and colleges [13]), for adolescents with type 1 diabetes on health literacy in the past 10 years. For this systematic review, the authors refer to the study of Bröder and colleagues, who identify [14] fourteen dimensions of health literacy that have been developed for children and adolescents, clustered in three core categories, namely (1) cognitive attributes, which correspond to the ability to think, learn and process information, (2) behavioural or operational attribute and (3) affective and conative attribute. Moreover, due to the broad term of digital health intervention in diabetes, the authors refer to the definition of the distal technologies, which included telehealth, mobile health, mHealth or messaging systems, mobile applications, game-based support, social platforms and patient portals [13].

The following questions will be addressed:

1. Which of Bröder and colleagues’s (2017) categories and dimensions are predominant when talking about health literacy in adolescents with T1DM?
2. Which distal digital health tools (Duke, 2018) are used for adolescents with T1DM for these categories and dimensions and how are they related?

## Methods

The PRISMA Statement and checklist (Preferred Reporting Items for Systematic Reviews and Meta-Analyses [15]) were adopted and followed. A protocol was published on PROSPERO (reg. NR: CRD42021282199) on 15 November 2021 and revision notes on 15 April 2022. An overview of the complete scoring procedure is available in the supplementary material.

### Eligibility criteria

The authors defined in advanced inclusion and exclusion criteria for this review. Studies were included in the review if they had: (1) T1DM adolescents population, (2) digital health interventions according to Duke and colleagues [13], respectively distal technologies include telehealth, mobile health (mHealth), game-based support, social platforms and patients portals, (3) health literacy according to Bröder’s definition [14], (4) studies reported in English, German or French, (5) original papers published in peer-reviewed journals, or reports published between 2011 and 2021 (6) articles from any country and setting (See Table 1).

**Table 1:** Eligibility criteria

Criterion	Inclusion	Exlusion
Time	January 2011 – October 2021	Studies before 2011 and after 2021
Language	English, German, French	Any other language
Type of publication	Original papers published in peer-reviewed journals or reports	Any non-original publication, any editorials, letters to editors, theses, books, abstracts
Focus of study	- Health Literacy according to Bröder’s definition (2017) - Digital Health Interventions according to Duke and colleagues (2018), respectively distal technologies include telehealth, mobile health (mHealth), game-based support, social platforms and patients portals	–
Study population	Articles including adolescents with Type 1 Diabetes Mellitus	Any other population
Setting	Any setting	–
Country	Any country	–

### Information sources

Electronic searches were performed in five databases in Medline (Medline, PubMed + via PubMed), The Cochrane Library, EMBASE (via Ovid), Web of Science (Wolters and Cluver) and PsycINFO from January 2011 to September 2021. The search took place between September and October 2021. In addition, grey literature searches were conducted in Google Scholar, OAlster and Trip. Furthermore, relevant studies that had been missed by electronic and hand-searching strategies were searched in the reference lists of all included studies. The authors updated the search in all databases on the 29th of December 2022 with no new relevant results according to the eligibility criteria.

### Search strategy

Based on the PICOS approach, synonyms and terms related to diabetes mellitus, adolescents, digital health interventions and health literacy were searched for relevant literature. The search strategy included a combination of English search terms: controlled vocabulary where applicable (e.g., Medical Subject Headings (MeSH) terms to search MEDLINE) and free vocabulary in titles and abstracts. Based on the block building approach, keywords and terms were combined using the Boolean operators AND and OR and were progressively checked for relevant hits. The search dates were informed for all the databases mentioned. The details of the search terms strategy of the different databases were mentioned. The search was restricted to the publication types of Systematic Reviews, Meta-Analysis, Clinical Trials, Randomized Control Trials and Qualitative Studies. Further restrictions on the date of publication and languages are mentioned above in the chapter eligibility criteria. Publications without abstract, pure abstract publications and non-procurable full texts were excluded. Regarding grey literature searches, the search strategy was undertaking with similar searches from the databases.

### Study selection process

All references captured by the search were uploaded to EndNote 20 (Clarivate Analytics; Philadelphia, PA, USA). After uploading all references and removing duplicates of the result of our search, two researchers (ANN and CW) were independently screened all titles and abstracts via the browser application Rayyan [16]. Records that were clearly not relevant were excluded. The two authors excluded records like conference abstracts, posters, letters to editors, etc. Disagreements over eligibility of studies were discussed and, if necessary, resolved by a third reviewer (VEA). Authors were contacted if clarification of their data or study methods were required. The process of data extraction was documented using the PRISMA Flow Diagram [15].

### Data collection process and data items

By using a standardised data collection form [17], the two reviewers (ANN and CW) extracted data independently from the included studies and compared them for discrepancies. Extracted data included: (1) reference/author (2) year (3) country (4) setting / study design (5) study population characteristic (6) methods (7) research question / study name (8) outcomes (9) study results (10) type of digital health intervention. The outcomes for each study were the following: (i) engagement (ii) communication with HCP (iii) metabolic control / glycemic control (iv) self-efficacy (v) quality of life (vi) HCP-Patient relationship (vii) collaboration with diabetes care team (viii) knowledge (ix) complication after education (x) participation and engagement (xi) user experiences related to patient empowerment (xii) conversational skills of moderators (xiii) internet use social networking online (xiv) self-management (xv) self-education (xvi) behaviour changes (xvii) psychological effect (xviii) efficacy of Social Network Sites (SNS).

### Study selection

**Fig. 1:**
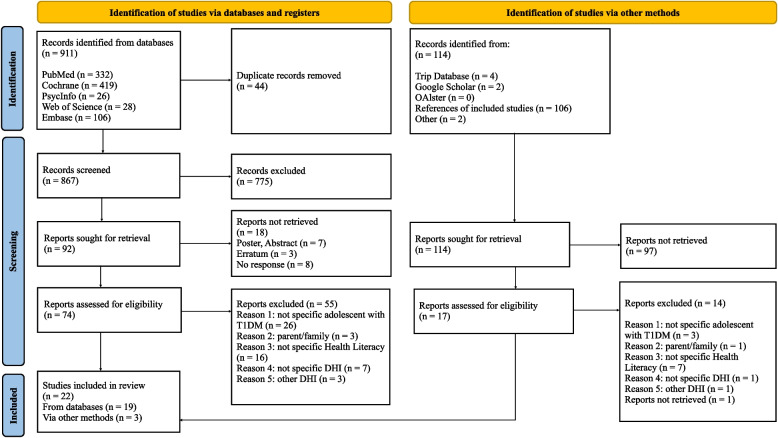
PRISMA Flow Diagram

From 911 records through the databases PubMed (*n* = 332), Cochrane (*n* = 419), PsycInfo (*n* = 26), Web of Science (*n* = 28) and Embase (*n* = 106), 44 duplicate records were removed and 867 titles and abstracts were screened. 775 records were excluded because of other types of diabetes, other special population (adults, old people), other diseases or since they were not related to this study. We identified 4.7% conflicts (41 articles) between the two authors. The differences had been discussed until an agreement was reached. Out of the 92 articles that had been included in the full text screening, 73 were excluded: reports not retrieved (*n* = 18) (poster or abstract (*n* = 7), erratum (*n* = 3), no response (*n* = 8), not specific adolescent with T1DM (*n* = 26), parents/families (*n* = 3), not specific Health Literacy according to Bröder’s definition (*n* = 16), not specific Digital Health Intervention (*n* = 7), other DHI (*n* = 3)). Following the identification of studies via other methods (grey literature), the authors identified 114 records through Trip Database (*n* = 4), Google Scholar (*n* = 2), OAlster (*n* = 0), references of included studies (*n* = 106) and other studies (*n* = 2). From 114 studies, 97 reports were not retrieved after abstract screening and 14 reports were excluded after full text screening: not specific adolescent with T1DM (*n* = 3), parent/family (*n* = 1), not specific Health Literacy (*n* = 7), not specific DHI (*n* = 1), other DHI (*n* = 1), reports not retrieved (*n* = 1). From all 1025 records (911 from the databases and 114 from other sources), 22 records (see Table 2) were included in the systematic review (19 from the databases and 3 from other sources). Three primary studies [6, 18, 19] are included in three secondary studies [5, 13, 20]. However, the authors decided to keep the studies to make the analysis more precise by enlarging the data set by all eligible and relevant data (See Fig. 1).

**Table 2:** Records included

	Authors and year	Title
Primary studies	Ayar et al. (2021) [21]	The Effect of Web-based Diabetes Education on the Metabolic Control, Self-efficacy and Quality of Life of Adolescents with Type 1 Diabetes Mellitus in Turkey
	Pembroke et al. (2021) [22]	Developing a video intervention to improve youth question-asking and provider education during paediatric diabetes clinic encounters: The Promoting Adolescents Communication and Engagement study
	Döğer et al. (2019) [23]	Effect of Telehealth System on Glycemic Control in Children and Adolescents with Type 1 Diabetes
	Malik et al. (2019) [24]	Adolescent Perspectives on the Use of Social Media to Support Type 1 Diabetes Management: Focus Group Study
	Sap et al. (2019) [25]	Effect of patient education through a social network in young patients with type 1 diabetes in a Sub-Saharan context
	Troncone et al. (2019) [26]	Psychological support for adolescents with type 1 diabetes provided by adolescents with type 1 diabetes: The chat line experience
	Vaala et al. (2018) [27]	Sharing and helping: predictors of adolescents’ willingness to share diabetes personal health information with peers
	Henkemans et al. (2017) [18]	Design and evaluation of a personal robot playing a self-management education game with children with diabetes type 1
	Frøisland & Årsand (2015) [6]	Integrating Visual Dietary Documentation in Mobile-Phone-Based Self-Management Application for Adolescents With Type 1 Diabetes
	Newton & Ashley (2013) [19]	Pilot study of a web-based intervention for adolescents with type 1 diabetes. Journal of Telemedicine and Telecare
	Nordfeldt et al. (2013) [28]	As facts and chats go online, what is important for adolescents with type 1 diabetes?
Secondary studies	Nkhoma et al. (2021) [29]	Digital interventions self-management education for type 1 and 2 diabetes: A systematic review and meta-analysis
	Zhao et al. (2021) [2]	Effectiveness of Internet and Phone-Based Interventions on Diabetes Management of Children and Adolescents With Type 1 Diabetes: A Systematic Review
	Rewolinski et al. (2020) [5]	Type I Diabetes Self-management With Game-Based Interventions for Pediatric and Adolescent Patients
	Duke et al. (2018) [13]	Distal technologies and type 1 diabetes management
	Chaves et al. (2017) [20]	Mobile applications for adolescents with type 1 diabetes mellitus: integrative literature review
	Swartwout et al. (2016) [30]	Use of Gaming in Self-Management of Diabetes in Teens
	Lazem et al. (2015) [31]	Games and Diabetes: A Review Investigating Theoretical Frameworks, Evaluation Methodologies, and Opportunities for Design Grounded in Learning Theories
	McDarby et al. (2015) [32]	An Overview of the Role of Social Network Sites in the Treatment of Adolescent Diabetes
	Dougherty et al. (2014) [33]	Telemedicine for Adolescents With Type 1 Diabetes
	Ho et al. (2014) [7]	Features of Online Health Communities for Adolescents With Type 1 Diabetes
	Pal (2014) [34]	Social Media for Diabetes Health Education—Inclusive or Exclusive?

### Study risk of bias assessment

To assess the methodological quality and minimise the risk of bias, the authors applied the 16 items revised instrument Amstar-2 [35] to systematic reviews and meta-analysis, ROBINS-I and RoB2 (the Cochrane’s risk of bias tools) for clinical trials and randomised control trials, the tool CASP, the Critical Appraisal Skills Programme checklist for quality appraisal for qualitative studies and NOS (Newcastle–Ottawa Scale) for longitudinal cross-sectional study [36].

### Risk of bias in studies

The quality was assessed by using the tools RoB2 Cochrane [20], ROBINS-I [37], NOS – Newcastle–Ottawa Scale [23], CASP – Critical Appraisal Skills Programme for primary research and Amstar-2 [21] for secondary research. Three RCT were assessed with the revised Cochrane risk-of-bias tool for randomised trials (RoB 2) [26, 35, 36]. The three Individually Randomized Group-Treatment (IRGT) Trials studies, grouped as intent-to-treat analysis (ITT) were judged as having some concerns (moderate risk). None of them was grouped as per-protocol (PP). The source used by the authors to help inform the risk-of-bias assessment was only the journal article with results of the trial. In the three studies, the domain (3) Risk of bias due to rising outcome data was assessed by the authors at low risk of bias. Regarding the domain (1) Risk of bias arising from the randomisation process, Newton and Ashley [36] and Ayar et al. [26] were judged at low risk-of-bias. The study of Henkemans et al. [35] was judged with some concerns because of missing information about the random and the concealed sequence allocation. Further, the baseline imbalances could suggest a problem. The domain (2) Risk of bias due to deviations from the intended interventions (effect of assignment to intervention), the domain (4) Risk of bias in measurement of the outcome and the domain (5) Risk of bias in selection of the reported result were assessed with some concerns in the three studies. Noticeably, the analysis intentions for all the studies were not available in the domain (5).

According to the assessment guidance from Sterne and colleagues [37], the study from Sap and colleagues [30] was judged at serious risk of bias because the authors assessed that the limitation of not providing an android phone could cause a bias due to confounding. This important confounding domain was not appropriately measured and controlled. However, the other domains were classified from the authors at moderate or low risk of bias: moderate risk of bias in selection of participants into the study (domain 2), low risk of bias in classification of intervention (domain 3), as well as due to missing data (domain 5) and in measurement of outcomes (domain 6). There was too little information to make a risk of bias judgement regarding the bias due to deviations from intended interventions (domain 4) and bias in selection of the reported result (domain 7).

The cohort study from Döğer and colleagues [28] was assessed with a score of 2 points out of 8, with no description regarding the assessment of outcome and the adequacy of follow up of cohorts, as well as the description of the derivation of the non-exposed cohort and the ascertainment of exposure.

The Critical Appraisal Skills Programme (CASP) tool was used for quality appraisal in qualitative evidence synthesis in six studies [12, 24, 27, 28, 31, 32]. The CASP tool does not produce results classified as overall ‘high’, ‘medium’ or ‘low’ quality [38]. All six studies were qualified as valuable (section C). In section B, the authors answered with ‘yes’ referring to the rigorousness of the data analysis as well as a clear statement of findings for all the six studies. All the studies have considered the ethical issues, except the study of Vaala and colleagues [31], responded by the answer’s option ‘can’t tell’. Regarding the section A, all studies have a clear statement of the aims of the research and an approbate recruitment strategy to the aims of the research (question 1 and 4). However, some concerns appear in section A. Indeed, in all the studies, it was not clear if the relationship between researcher and participants have been adequately considered (only the answer’s options ‘no’ or ‘can’t’ tell have been used by the reviewers).

The tool Amstar-2 was used for the assessment of the 11 secondary data studies [5, 8, 11, 17, 22, 29, 33, 34, 39–41]. The authors assessed one study as high-quality review [41] with no critical weakness, which provide an accurate and comprehensive summary. Two reviews were evaluated as low quality [8, 11], which means that the reviews should have a critical flaw. The quality of the reviews is not sufficient in 8 out of 11 studies, which implies, according to Shea et al. [21], that the reviews have ‘more than one critical flaw and should not be relied on to provide an accurate and comprehensive summary of the available studies’ (See Table 3).

**Table 3:** Assessment tools and results of the critical appraisal for included studies

	Authors and year	Assessment tools	Methodological Quality Scores
Primary studies	Pembroke et al. (2021) [22]	CASP	-
	Ayar et al. (2021) [21]	RoB2.0 Cochrane	Some concerns
	Döğer et al. (2019) [23]	NOS	-
	Malik et al. (2019) [24]	CASP	-
	Troncone et al. (2019) [26]	CASP	-
	Sap et al. (2019) [25]	ROBINS-I Cochrane	Serious risk of bias
	Vaala et al. (2018) [27]	CASP	-
	Henkemans et al. (2017) [18]	RoB2.0 Cochrane	Some concerns
	Frøisland & Årsand (2015) [6]	CASP	-
	Newton & Ashley (2013) [19]	RoB2.0 Cochrane	Some concerns
	Nordfeldt et al. (2013) [28]	CASP	-
Secondary studies	Nkhoma et al. (2021) [29]	Amstar-2	High quality review
	Zhao et al. (2021) [2]	Amstar-2	Low quality review
	Rewolinski et al. (2020) [5]	Amstar-2	Low quality review
	Duke et al. (2018) [13]	Amstar-2	Critically Low quality review
	Chaves et al. (2017) [20]	Amstar-2	Critically Low quality review
	Swartwout et al. (2016) [30]	Amstar-2	Critically Low quality review
	Lazem et al. (2015) [31]	Amstar-2	Critically Low quality review
	McDarby et al. (2015) [32]	Amstar-2	Critically Low quality review
	Pal (2014) [34]	Amstar-2	Critically Low quality review
	Ho et al. (2014) [7]	Amstar-2	Critically Low quality review
	Dougherty et al. (2014) [33]	Amstar-2	Critically Low quality review

### Synthesis methods

For the strategy for data synthesis, the authors provided a qualitative synthesis of the results to summarise the evidence. To recognise which dimensions of health literacy (as described by Bröder and colleagues [14]) were most present, the authors reported each time an outcome matched one of Bröder and colleagues' definitions in the 22 studies selected for analysis. Specifically, a significant improvement in outcomes corresponding to the definitions of the dimensions by digital interventions (according to Duke and colleagues [13]). Each dimension treated in the analysed studies (one or several) was mentioned indicating the digital tool applied for the indicated dimension.

## Results

### Study characteristics

Out of a total of 22 articles included in this review, 11 articles were primary studies [6, 18, 19, 21–28] and 11 secondary studies [2, 5–7, 13, 20, 30–34]. The study design of the primary studies were randomised controlled trials – RCTs (3), non-randomised controlled clinical trial (1), qualitative studies (5), longitudinal cross-sectional study (1), quantitative study (1). Regarding the study design of the secondary studies, two were systematic reviews, five literature reviews, two narrative review and two brief reviews. The articles were developed and/or published in the following countries: United States of America – USA (8), Ireland (2), Turkey (2), United Kingdom – UK (2), Brazil (1), Cameroon (1), China (1), Italy (1), Netherlands (1), Norway (1), Sweden (1), Taiwan (1). In the primary studies, the sample sizes ranged from 12 to 161 with an average of 61 and concern only a population of patients with type 1 diabetes. The age range was from 2 to 26 years. All the primary studies included the age of 13 to 17. One study has a lower bound of 2 [25] and another study has an upper bound of 26 [30]. The studies lasted between 3 and 10 months. Concerning the secondary studies, all the studies are related to adolescents with diabetes, more than half of the studies are addressed to a population of patients with type 1 diabetes only, the other studies apply to a population of type 1 and type 2. The distribution of all included articles according to year of publication was the following: 2021 (4), 2020 (1), 2019 (3), 2018 (2), 2017 (3), 2016 (1), 2015 (3), 2014 (3) and 2013 (2).

Regarding the methods, three of the five qualitative studies were focus groups and four studies were interviews. Most of the measurements were based on quality of life (QoL), self-efficacy, communication features, social support, conversations skills, diabetes knowledge and willingness to share information. Most of the outcomes examined were as follows: knowledge, communication and relationship with HCP, self-efficacy, quality of life and engagement (See Table 4).

**Table 4:** Study characteristics

Author Year Country	Setting—Study design	Study population characteristic	Methods	Research question	Outcomes	Study Results
Pembroke et al. 2021 Ireland [22]	Qualitative study (Focus group, One-to one interviews, Two workshops)	*n* = 13 T1DM participants ages 11 to 17 years. *n* = 14 parents *n* = 7 HCP 3-months study	3 FG 24 Interviews 2 Workshops Topic guide, demographic survey Interative development of a video	Developing a video intervention to improve youth question-asking and provider education during paediatric diabetes clinic encounters: The Promoting Adolescents Communication and Engagement study	1. Engagement 2. Communication with HCP	1. Message of empowerment 2. Important role of clinic visits and HCP 3. Promoting independence 4. Communication with HCP and Engagement through reassurances 5. Development of trust and relationships 6. Practical advice on preparing questions
Ayar et al. 2021 Turkey [21]	RCT	*n* = 62 T1DM participants ages 12 to 18. 6-months study	E: *n* = 30 C: *n* = 32 E: Web-based diabetes education Tools: powerpoint, quizzes and blogs. 2 × per week to update the blog, write comments or share experiences C: Diabetes education in clinical setting (tools: pamphlets and/or posters) Measurements: HbA1c Level QoL Self-efficacy scale	The Effect of Web-based Diabetes Education on the Metabolic Control, Self-efficacy and Quality of Life of Adolescents with Type 1 Diabetes Mellitus in Turkey	1. Metabolic control 2. Self-efficacy 3. QoL	Metabolic control A1C: no significant difference between E and C (*P* > 0.05) Self-efficacy and QoL: statistically significant difference between E and C
Döğer et al. 2019 Turkey [23]	Observational study (Longitudinal cross-sectional study)	*n* = 82 T1DM participants ages 2 to 18 8-months study	Measurements based on: Communication features People wo contacted the HCP Call frequency HCP members consulted Counselling topics HbA1c Level	Effect of Telehealth System on Glycemic Control in Children and Adolescents with Type 1 Diabetes	1. Patient – HCP relationship and communication	Communication features used: Whatsapp: 57.3% Phone: 29.3% Short message service: 13.4%
Malik et al. 2019 USA [24]	Qualitative study (Focus group)	*n* = 45 T1DM participants ages 13 to 19 years 3-months study	5 Focus group with 8 to 19 participants per group 110 to 120 min Semistructured questions Qualitative content analysis Emergent themes in 4 domains: acceptability, demand, implementation, practicality	Adolescent Perspectives on the Use of Social Media to Support Type 1 Diabetes Management: Focus Group Study	1. Collaboration with diabetes care team	Domain 1: Acceptability and demand 1. Improved communication outside of clinic visits 2. Independence in diabetes Self-Management 3. Delivery of timely and personalised diabetes care 4. Connection to other youth with diabetes Domain 2: Implementation and practicality 5. Ensure patient privacy 6. Maintain Provider-Patient relationship 7.Recognise that social media is not currently used
Troncone et al. 2019 Italy [26]	Qualitative study	*n* = 161 T1DM participants ages 12 to 18 years 10-months study	Session of chat conversation with specific topic 60 min Analysis with two coding schemes: social support (all messages) and conversations skills (messages written by moderators)	Psychological support for adolescents with type 1 diabetes provided by adolescents with type 1 diabetes: The chat line experience	1. Examining the extent to which posted messages provide social support 2. describing qualitatively the social support provided 3. describing quantitatively and qualitatively the conversational skills of moderators	37 chat sessions during study period, 17 025 individual posts (10 735 by participants, 6290 by moderators) Topics: management of the disease, diabetes-related problems, nutrition, and the emotional impact of diabetes Emotional support: Participants: 79.97%, Moderators: 34.56% Information support: Participants: 16.21%, Moderators: 52.89%
Sap et al. 2019 Cameroon [25]	Non-randomized controlled clinical trial	*n* = 54 T1DM participants ages 13 to 26 years 6-months study	E: *n* = 25 C: *n* = 29 FG, weekly session of 60 to 90 min for 4 weeks Themes: Definitions of diabetes, insulin, blood glucose objectives, HbA1C; short- and long-term complications; the use of insulin and self-monitoring of glycemia; diet Measurements: - Knowledge questionnaire	Patient education through social network helped to improve knowledge on T1DM and to reduce acute complications without an improvement of glycemic	Evaluation: Glycemic control Knowledge Complications after education	Knowledge: Results increase significantly in E. (*P* < 0.01). Results decrease in C. (*P* = 0.33) Acute complications decrease in E (*P* = 0.46) and increase in C. (*P* = 0.01) No improvement in HbA1c in E and C
Vaala et al. 2018 USA [27]	Quantitative Study (Online Survey)	*n* = 134 T1DM participants ages 12 to 18 years	Measurements: Glycemic control Experience to help Perception about social resources Sharing and helping beliefs Willingness to share information Statistical analyses: SPSS v23	Sharing and helping: predictors of adolescents’ willingness to share diabetes personal health information with peers	Sharing information and helping others: Participation Engagement	Participants: willingness to share personal health information with peers (6 of 10 types) Particularly receptive to share about diabetes tasks Less willing to share information with others wenn poorer glycemic control
Henkemans et al. 2017 Netherlands [18]	RCT	*n* = 27 T1DM participants ages 7 to 14 years 18-weeks study	3 Sessions: C: *n* = 11 E1: *n* = 9 (personal robot) E2: *n* = 7 (neutral robot) Measurements: SCI: Self-care inventory HrQoL: Health related quality of life SDT: determinants of self-determination CRI: Child-robot interaction DK: Diabetes knowledge Motivation, fun	Design and evaluation of a personal robot playing a self-management education game with children with diabetes type 1	1. "Learning by playing with a robot” 2. Effects of personalisation on child-robot interaction in a clinical setting	Knowledge: Results increase in E1 and E2 but not in C. *(P* = *.001)* SDT: higher score in E1 than E2 (*P* = .02) Pleasurable (*P* = .04), more questions correctly answered (*P* = .02), more motivation to play a fourth time (*P* = .03) with the robots More engagement, attention, more social and positive with E1 (P < .05)
Frøisland & Årsand 2015 Norway [6]	Qualitative study (Semistructured interview)	*n* = 12 T1DM participants ages 13 to 19 years 3-months study	Introduction of a smart-phone and 2 software application Semistructured interviews after 3 months 30 to 60 min Deductive approach based on empowerment theory Predefined empowerment factors: Comprehension and management Meaning of life and influence Recognition (self-treatment and self-medication) Coping experience (include access and ability to exploit knowledge, social resources or equipment) Changes to improve the situation	Integrating Visual Dietary Documentation in Mobile-Phone-Based Self-Management Application for Adolescents With Type 1 Diabetes	1. User experiences related to patient empowerment	Empowerment factors: Improvement in comprehension and feeling of managing the self-treatment Increasing social acceptance Positive feedback and SMS solution to give a sense of „being in charge“ Improvement in knowledge and understanding Integration of knowledge and resources to take rational decisions Ability to evaluate the effectiveness of decisions
Newton & Ashley 2013 USA [19]	RCT (Pilot Study)	*n* = 59 T1DM participants ages 13 to 18 years 7-weeks study	Mixed model design E: log in encouraged 3x/week Measurements: QoL for Youths Self-efficacy Outcome expectations Statistical analyses: SPSS	Pilot study of a web-based intervention for adolescents with type 1 diabetes	1. QoL for Youths 2. Self-efficacy 3. Outcome expectations	QoL: no significant differences between E and C (*P* = 0.63*)* Self-efficacy*:* no significant differences between E and C (*P* = 0.53) Negative Outcome Expectations (*P* = 0.31)
Nordfeldt et al. 2013 Sweden [28]	Qualitative study (Focus group)	*n* = 24 T1DM participants ages 10 to 17 years	8 Focus group 60 to 90 min Qualitative content analysis Questions focused on the participants’ experiences and need for contact with other adolescents with T1DM	As Facts and Chats Go Online, What Is Important for Adolescents with Type 1 Diabetes?	To understand: 1. Information-seeking behaviour 2. Internet use 3. Social networking online	3 main categories: 1) Aspect of Security 2) Updating 3) Plainness Sub-categories: 1) seriousness, integrity and identity; 2) news value, facts and eye-catching; 3) layout, content and congeniality
Nkhoma et al. 2021 Taiwan [29]	Systematic Review and Meta-Analysis	T1DM and T2DM Range age: 13 – 70	Inclusion criteria: 2010 – 2019 RCTs in English T1DM or T2DM DHI: mobile health, social media or web-based (e-health) Measurements: Glycemic control, HrQoL, Knowledge	Digital interventions self-management education for type 1 and 2 diabetes: A systematic review and meta-analysis	Impact on: 1. Metabolic Control (HbA1c) 2. Diabetes knowledge 3. HrQoL	35 of 4295 articles identified 1. Mobile Health: Mobile Apps and text messages: Mobile Apps: Educational materials, Diabetes education and support Text messages: Information or motivation; involving dialogue with HCP about educational information 2. Web-based (e-health): educational and motivational information. Portals to facilitate dialogue with HCP 3. Social media: education, self-management, medical adherence, coping and problem-solving
Zhao et al. 2021 China [2]	Systematic Review	Children and Adolescents with T1DM < 20 years	Inclusion criteria: 1989 – 2020 RTCs in English and Chinese DHI: phone call, text message, web, application or app, telemedicine, Skype or other social media	Effectiveness of Internet and Phone-Based Interventions on Diabetes Management of Children and Adolescents With Type 1 Diabetes: A Systematic Review	Impact of: 1. Metabolic control 2. Self-management behaviour changes 3. Psychological effect	23 of 780 articles identified Non-significant improvement in adherence (behaviour changes) Non-significant improvement in QoL Improving metabolic control Improving Self-efficacy
Rewolinski et al. 2020 USA [5]	Literature Review	Children and Adolescents with T1DM	Inclusion criteria: 2010 – 2018 T1DM Peer-reviewed DHI: Game-based intervention (Serious Game, Gamification) Evaluation/outcomes: knowledge, behaviour or engagement	Type I Diabetes Self-management With Game-Based Interventions for Pediatric and Adolescent Patients	Impact on: 1. Knowledge 2. Behaviour 3. Engagement	9 of 217 articles identified Serious Games: knowledge outcomes and engagement Gamification and Serious Game (combination): behavioural outcomes and engagement
Duke et al. 2018 USA [13]	Literature review with narrative synthesis	Adolescents and adults with T1DM	Inclusion criteria: 2005 – 2016 T1DM DHI: Distal technologies	Distal technologies and type 1 diabetes management	Distal technologies to improve T1DM Management: 1. Identification about effectiveness 2. Highlighting potential complications 3. Identification of barriers	80 of 10,325 articles identified Distal technologies: telehealth, mHealth, game-based support, social platforms, patient portals
Chaves et al. 2017 Brazil [20]	Integrative Literature Review	Adolescents with T1DM	Inclusion criteria: 2012 – 2017 Application for Adolescent with T1DM	What are the features in mobile applications for self care of adolescents with type 1 diabetes mellitus reported on in the literature?	Features of mobile application for self-care	12 of 248 articles identified Social relationships: Health professionals (message sending) Peers (chat rooms) context
Swartwout et al. 2016 USA [30]	Literature Review	Adolescents with T1DM and T2DM	Inclusion criteria: 2001 – 2016 T1DM or T2DM DHI: Serious Game Self-care, Self-management	Use of Gaming in Self-Management of Diabetes in Teens	Examination of the current gaming application Impact on: 1. Knowledge 2. Self-efficacy 3. Communication 4. BG monitoring (Metabolic control)	11 articles identified - BG Monitoring: 4 studies showed improvement - Knowledge (diabetes-related): 4 studies showed improvement
Lazem et al. 2015 UK [31]	Brief Review	Children and Adolescents with T1DM and T2DM	Inclusion criteria: 2010 – 2015 English-language OHCs for T1DM Design and/or evaluation of games	Games and Diabetes: A Review Investigating Theoretical Frameworks, Evaluation Methodologies, and Opportunities for Design Grounded in Learning Theories	1. Understanding of the current landscape of digital games 2. Usability 3. Impact of well-established learning theories	18 of 35 articles identified Discussion of 3 examples: content, theoretical framework, methods to evaluate 7/18: educational topic related to diabetes
McDarby et al. 2015 Ireland [32]	Narrative Review	Adolescents with DM	NR	An Overview of the Role of Social Network Sites in the Treatment of Adolescent Diabetes	1. Evidence of the efficacy of SNS	Lack of evidence: - Knowledge improvement: increasingly debated and few evidence - Health and behavioural outcomes often not examined - Lack of control misinformation
Pal 2014 UK [34]	Narrative Review	NR	NR	Social Media for Diabetes Health Education—Inclusive or Exclusive?	Highlighting the issues of using social media Impact of: 1. Self-education 2. Self-management 3. Education for staff	Advantages: deep learning, active involvement, involving cognitive skills, active participation in discovering knowledge, ‘Peer learning’, platform for self-education Disadvantages: need a high level of health literacy
Ho et al. 2014 USA [7]	Brief review	Adolescents with T1DM	Inclusion criteria: (Website) Search in March 2013 English-language OHCs for T1DM	Features of Online Health Communities for Adolescents With Type 1 Diabetes	OHCs: 1. Identification and characterisation of features	18 of 50 (Search hits of more than 22 million results) OHCs analysed Social learning and networking (forum or discussion board, one-to-one communication via private messaging, sharing personal stories) Information (staff-written articles, blog, email newsletter, videos, product reviews) Guidance („Ask the experts“ Engagement (gamification, social recognition) Personal health data sharing (tools for monitoring own health data)
Dougherty et al. 2014 USA [33]	Brief review	Adolescents with T1DM 13 – 18 years	Inclusion criteria: 2006 – 2013 In English, available online 13 – 18 years old	Telemedicine for Adolescents With Type 1 Diabetes	1. Define telemedicine intervention to be used for T1DM 2. Impact of telemedicine interventions on diabetes control	15 of 90 articles identified Communication with provider Education between clinic visits

*DM* Diabetes Mellitus, *T1DM* Type 1 Diabetes Mellitus, *T2DM* Type 2 Diabetes Mellitus, *NR* Not Reported, *DHI* Digital Health Interventions, *HbA1c* Glycosylated Hemoglobin, *HrQoL* Health-Related Quality of Life, *SCI* Self-Care Inventory, *RCT* Randomised controlled trial, *E*: Experimental Group, *C* Control Group, *HCP* Healthcare Provider, *FG* Focus Group, *BG* Blood Glucose

### Health literacy in childhood and youth: definitions and models from Bröder and colleagues

In order to identify the category and dimensions of Bröder and colleagues [14], the authors extracted 43 items corresponding to a positive impact of health literacy by using a digital health intervention. The second category (behavioural or operational attribute) is the most common with 48.8% of the cases, followed by the first category (cognitive attributes) with 27.9% and the third category (affective and conative attribute) with 23.3%.

According to the extract of definitions corresponding to the dimensions of health literacy defined by Bröder and colleagues, the dimension (7) Communication and interaction is the one that occurs the most, at 25.6% in 11 studies [6, 7, 13, 20, 22–24, 27–29, 33]. The most prevalent digital health interventions that correspond to the concept of distal technologies [13] are social platforms (including social media). The digital health intervention which appears mostly in the 11 studies selected for the review are social platforms (included social media), according to the definition of distal technologies [13]. Telehealth such as phone, SMS, WhatsApp, but also mobile applications are also applied. The two interventions that are not involved in this dimension are game-based support and patient portal. The second most frequently mentioned dimension in the 22 studies analysed is the dimension (1) knowledge with 18.6% in eight studies [5–7, 13, 18, 25, 29, 33]. The digital intervention game-based support appears twice, once as a robot, once as gamification and serious game, and every intervention mentioned by Duke and colleagues [13] are present, except the intervention patient portal. The third largest dimension in the studies selected is the (14) interest and motivation with 14% in 6 studies [5–7, 18, 22, 29]. The digital health interventions mentioned in those studies are a video intervention, game-based support (twice), mobile application, social platforms (online health community) (See Table 5).

**Table 5:** Bröder and 3 dimensions

Study	Dimension 7: communication and interaction	Dimension 1: knowledge	Dimension 14: interest and motivation	Telehealth (telephone calls or videoconferencing)	mHealth	Mobile application	Game-based support	Social platforms (included social media)	Patient portals	Others
Pembroke et al. (2021) [22]										Video intervention
Ayar et al. (2021) [21]										
Döğer et al. (2019) [23]				Telehealth (phone)	SMS, Whatsapp					
Malik et al. (2019) [24]								Snapchat Facebook Instagram Twitter YouTube		
Troncone et al. (2019) [26]										
Sap et al. (2019) [25]					Whatsapp					
Vaala et al. (2018) [27]								Social media (online communities)		
Henkemans et al. (2017) [18]							Robot			
Frøisland & Årsand (2015) [6]						Mobile application				
Newton & Ashley (2013) [19]										
Nordfeldt et al. (2013) [28]								Social networking online		
Nkhoma et al. (2021) [29]										Mobile health, web-base, social media
Zhao et al. (2021) [2]										
Rewolinski et al. (2020) [5]							Serious game and gamification			
Duke et al. (2018) [13]										Not specific
Chaves et al. (2017) [20]						Mobile application				
Swartwout et al. (2016) [30]										
Lazem et al. (2015) [31]										
McDarby et al. (2015) [32]										
Pal (2014) [34]										
Ho et al. (2014) [7]								Online Health Community		
Dougherty et al. (2014) [33]				Telehealth						

### Qualitative synthesis

11 studies [6, 7, 13, 20, 22–24, 27–29, 33] have highlighted the importance of communication and interaction between HCP and patients. More precisely, Pembroke et al. [22] concluded that patient engagement and communication increases through reassurance. Patients feel more comfortable talking and asking questions when a relationship and trust has been established with the HCP. Döğer et al. [23] concluded that Instant Messaging was the social media that patients prefer to use to communicate with HCPs. Malik et al. [24] also concluded that social media improve communication outside of clinic visits and allows for more open communication. Beyond that, they enable a closer relationship with HCP and better understanding of personal life. Vaala et al. [27] also highlights the importance of communication through social media sharing personal health information with peers. Frøisland & Årsand [6] show that mobile applications based on visualisation bring a sense of recognition through positive feedback. According to Nordfeldt et al. [28], the use of online social networking is effective if professionals are behind the site: it increases the importance of security through trustworthiness and reliability and the importance of confidence in relationships for maintaining seriousness, integrity and identity. Nkhoma et al. [29] emphasise dialogue with HCP on educational information. Duke et al. [13] distinguish two types of communication between patients and HCP, the synchronous (facilitated by telehealth) and the asynchronous (facilitated by mHealth). The authors also highlight patient portals for sharing of personal health records and other tools. As for Chaves et al. [20], the authors conclude a strengthening of social relationships with HCP through messaging and with peers through chat rooms. Several results of the selected studies also underline the importance of cooperation with other Peers and emotional support [13, 22, 24, 26, 27].

The authors found contradictions, particularly concerning the results on self-efficacy and quality of life. Indeed, Zaho et al. [2] and Ayar et al. [21] conclude an improvement of self-efficacy, which was not observed by Newton & Ashley [19], whereas it is the same digital intervention tool (Website) for Ayar et al. [21] and Newton & Ashley [19]. The other discrepancy concerns quality of life, which Ayar et al. [21] concluded had a significant difference, but Zhao et al. [2] and Newton & Ashley [19] did not. Although the outcome of metabolic control was not considered in this review, the authors still point out discrepancies in the effectiveness of distal digital tools on this outcome: some studies show a significant difference or improvement in metabolic control (e.g. Zhao et al. [2]), while others conclude that there is no significant difference (Ayar et al. [21], Sap et al. [25]). However, the digital interventions are different, which make the comparison difficult.

## Discussion

The intent of this systematic review was to provide an overview of the literature on the current evidence base regarding the impact of digital health interventions for adolescents with T1DM on health literacy in the past ten years. Furthermore, the authors evaluated the quality of the reviews. They employed rigorous methodologies to identify relevant articles answering their research question. The revised PRSIMA (The Preferred Reporting Items for Systematic reviews and Meta-Analyses) 27 item checklist [15] were used by the authors. Adolescents with type 1 diabetes face a multitude of challenges. These challenges can be supported by digital tools of two types: distal and proximal. Proximal digital tools such as insulin pumps and continuous glucose monitoring devices have become more widespread in recent years to facilitate and improve the management of type 1 diabetes. However, it is important to note that not all patients have the same access to these proximal digital tools and that this access depends mainly on the treating HCP, the insurance coverage and the care structures—which underlines inequalities in care more generally. One of the main inequalities lies in the socio-economic status of the patient, that directly influences the extent to which it is covered by health insurance. The same applies to the 'distal' digital tools on which the authors focused. First, this study showed us that the effectiveness of their use was limited for several reasons. For a start, these tools must be be introduced most of the time by the treating doctor or HCP and their use must be followed and encouraged by them. With the lack of consistency in the evidence, the bewildering variety of choices of different distal digital tools and probably the lack of time and affinity for them, the use of these tools is probably not employed to its full potential. In addition, these tools are even less recognised by health insurance companies and are mostly unknown to adolescents. Moreover, faced with the multitude of possibilities, the patient may be confused about the wide choice, veracity and reliability of the tools. The commercial influences of these tools are also to be taken into consideration. Furthermore, in Germany for example, at national level, the development of DiGA (digital medical device of risk class I or IIa according to MDR, the medical device regulation or, in the framework of the transitional provisions, according to MDD, the medical devices directive) [39] still has very little to do with the management of type 1 diabetes and it should also be used in conjunction with the healthcare provider (i.e. even if more and more DiGA were on the market, patients or HCPs would still have to be aware of and willing to use them). As for the existing free applications, most are not specifically applied to the type 1 adolescent group and are therefore not adequate to meet the specific demands of this population. For example, Sun and colleagues [40] demonstrate in their study that the effectiveness of mobile applications differs between type 1 and type 2. Furthermore, the multitude of definitions regarding 'distal' technologies vary considerably and while some studies show an improvement in HL, others show no significant improvement [30–32]. Additionally, 'distal' digital tools are used for secondary prevention and not primary prevention, as is the case for 'proximal' digital tools. These tools should not minimise the importance of face-to-face intervention, but be used as a complementary tool, as a mediator to strengthen the HCP-Patient relationship and interaction [41]. Finally, it should not be assumed that all adolescents necessarily have access to a smartphone. Hence, there is a strong need for individualised care and investigation of the socio-economic situation, the commitment of the patient, their clinical and behavioural characteristics which may influence the effectiveness of the tools used [10]. Secondly, this study allows us to raise some questions and criticisms regarding the term Health Literacy, which, although already defined for the first time in the 1970s by Simonds [37] and taken up by the WHO in 1998, has in recent years undergone many evolutions and contradictions between concepts, definitions and models [38]. The international definition and model still regularly cited in the scientific world is that of Sørensens [42], although it is now widely discussed. Many sub-themes revolve around HL and can be confusing, while defining it precisely. Indeed, themes such as education, self-management, patient-management, communication, adherence, motivation, emotional health, relationship, self-awareness, empathy, quality of life, etc. are sometimes used to define HL, other times to express its causes or consequences. The instrument for measuring HL, the European Health Literacy-Survey (HLS-EU), developed at European and national level (HLS-GER 2) [43], is widely criticised by Steckelberg and colleagues [44] for several reasons, including that of measuring health competencies by limiting themselves to personal competencies. The principle of the value of subjective assessments is also questioned, especially regarding the issue of objectivity. Another criticism is that only health knowledge and functional HL are measured, and not interactive and critical HL, three dimensions developed by Nutbeam [45]. Because of these criticisms, the measurement of HL as an outcome was not adopted by the authors of the present study. Consequently, the authors of this systematic review relied on more comprehensive, detailed and appropriate definitions for adolescents, such as proposed by Bröder and colleagues [14]. In a recent study by Schulenkropf and colleagues [46], in which an analysis of interviews with experts from 32 countries regarding the definition of HL was made, the authors concluded that the addictions, ages and developmental stages of a specific group should be considered. Indeed, the records analysed of this study do not take into account inequalities and what influence they exert on groups and persons, e.g. education level (issue of literacy and illiteracy), low social status or a history of immigration, and in particular people with personal experience of immigration. Thus, the studies included in the review showed that the dimension of communication and interaction (dimension defined by Bröder and colleagues [14]) was the most mentioned, a statement supported by other studies [23, 47–49] that demonstrate the importance of simple and quick contact with HCPs, an HCP-patient relationship, individualised care, personalised, direct and regular feedbacks for useful individual information, which, as a result, also allows reaching a higher percentage of the population [47]. This continuous follow-up and participation of patients allows for better empowerment and self-management of the disease. Frøisland and Årsand [6] warns that this individualised relationship can lead to a situation, where HCP put more weight on their own instead of the patients’ goals, thereby increasing the divergence of both parties. Hence the importance of the patient-centred (PC) principle defined McCance et al. [50] among others (originally from the field of care), which focuses on treating people as individuals, building trust and mutual understanding and developing a positive relationship. In 2015, the World Health Organisation [51] also developed a global strategy for integrated and person-centred health services. Studies by Scholl and colleagues [52] and Zeh and colleagues [53] also demonstrate the need for a good HCP-patient communication, HCP-patient relationship, patient as unique person but also for a better integration of medical or non-medical care through the included patient. Putting the individual at the centre incorporates the socio-economic background and resources in order to better understand inequalities, but also draw the attention to the needs and desires of the individual patients. Hower and colleagues [54] and Leidner and colleagues [55] refer to patient-centred care (PCC) and identify system-level determinants associated with the implementation of PCC and highlight inter-organisational collaboration and information sharing as facilitators of PCC, enabling seamless cycles of care for patients. The study shows a need for a model change at the system and external structure level, from disease-centred to a patient-centred approach, 'aligning policy and reimbursement decisions with patient needs and values' [55].

## Conclusion, limitations and future research

Our systematic review has some limitations that need to be taken into account when interpreting the results. First, it is possible that not all MESH terms used were incorporated. Furthermore, we limited our search to published articles and restricted the search to five database sources, three languages (English, German and French) and the last ten years, which could imply a potential risk of bias of publication. It is also possible that some relevant publications were overlooked, especially for studies dealing with type 2 diabetes or different populations, the results of which might also have been relevant for the systematic review. Secondly, the studies included in this systematic review had different characteristics, including a wide range of outcomes, which makes it difficult to make a clear and high-quality comparison. This heterogeneity may influence the reliability of our results. In addition, some of the studies reviewed have limitations such as limited time and small population size. Thirdly, the authors used very precise definitions: the definition of Bröder and colleagues for HL and the definition of Duke and colleagues for digital health interventions. Extending the definitions could have influenced the results. Fourth, most of the studies included did not provide robust evidence, which could influence the results of the analysis. Of the 22 studies, only one had low risk of bias.

An initial objective of this systematic review was to identify Bröder and colleague’s (2017) categories and dimensions when talking about health literacy in adolescents with type 1 diabetes. The results of this study show the importance of communication and interaction between HCP and adolescents patients with T1DM. The second question in this study sought to determine which distal digital health tools (Duke, 2018) are used for adolescents with T1DM for these categories and dimensions and how are they related. The limitation to determine the relation between HL and digital form of health care technology is important. This study did not find a significant answer to the questions because of the lack of consistent studies. However, this broad overview allows to give a direction towards further research, innovations and optimisation that are eagerly needed and therefore recommended to explore the potential and efficacy of digital health interventions in optimising the communication and interaction between HCP and patients, which can be used to support and complement face-to-face interactions between the two parties.

**Table:** 

**LINKING EVIDENCE TO ACTION:**
• Health Literacy skills needs to be enhanced, especially the dimension of communication and interaction between HCP and adolescent patients with T1DM. This should be taken seriously in research and clinical practice
• Social media have potential benefit to increase communication and interaction between HCP and adolescent patients with T1DM
• Interaction, communication and relationship with peers are important but with accompaniment of professional
• The distal digital health interventions play an important role in Health Literacy for adolescents with T1DM, but do not replace the face-to-face interaction with HCP

### Protocol and registration

The registration number of this review in the PROSPERO register is CRD42021282199. The registration has been submitted on the 15th of October 2021 and published the 15th of November 2021. Changes were reported on the 15th of April 2022.

## Supplementary Information

**Additional file 1: Appendix 1.** Full search strategy. **Appendix 2.** Scoring information – Results of the critical appraisal using the RoB2 tool. **Appendix 3.** Scoring information – Results of the critical appraisal using the ROBINS-I. **Appendix 4.** Scoring information – Results of the critical appraisal using the NOS. **Appendix 5.** Scoring information – Results of the critical appraisal using the CASP. **Appendix 6.** Scoring information – Results of the critical appraisal using the Amstar-2 tool.  

## Data Availability

The datasets used and/or analysed during the current study available from the corresponding author on reasonable request.
