# Supplementary material for: Impact of digital health interventions for adolescents with type 1 diabetes mellitus on health literacy: a systematic review
Source: BMC Endocr Disord. 2023 Mar 31;23:70. doi: 10.1186/s12902-023-01321-6 (PMC10064727; doi:10.1186/s12902-023-01321-6)
Supplement: Supplementary file 1 — Additional file 1: Appendix 1. Full search strategy. Appendix 2. Scoring information – Results of the critical appraisal using the RoB2 tool. Appendix 3. Scoring information – Results of the critical appraisal using the ROBINS-I. Appendix 4. Scoring information – Results of the critical appraisal using the NOS. Appendix 5. Scoring information – Results of the critical appraisal using the CASP. Appendix 6. Scoring information – Results of the critical appraisal using the Amstar-2 tool. [file 12902_2023_1321_MOESM1_ESM.pdf]

SUPPLEMENTARY MATERIAL

Appendix 1: Full search strategy

Appendix 2: Scoring information – Results of the critical appraisal using the RoB2 tool

Appendix 3: Scoring information – Results of the critical appraisal using the ROBINS-I

Appendix 4: Scoring information – Results of the critical appraisal using the NOS

Appendix 5: Scoring information – Results of the critical appraisal using the CASP

Appendix 6: Scoring information – Results of the critical appraisal using the Amstar-2 tool

Appendix 1: Full search strategy

PubMed_Date Run: 05.10.2021, 10:38:17

Search	Query	Items found
#52	("adolescent*"[Title/Abstract] OR "adolescent*"[MeSH Terms] AND "diabetes mellitus"[Title/Abstract] OR "diabetes mellitus"[MeSH Terms]) AND ("health literacy"[Title/Abstract] OR "self management"[Title/Abstract] OR "self management"[MeSH Terms] OR "disease self management"[Title/Abstract] OR "self management of diabetes"[Title/Abstract] OR "self management education*"[Title/Abstract] OR "patient participation*"[Title/Abstract] OR "patient participation*"[MeSH Terms] OR "health education*"[Title/Abstract] OR "health behavior*"[Title/Abstract] OR "health behavior*"[MeSH Terms] OR "health knowledge*"[Title/Abstract] OR „motivation*"[Title/Abstract] OR „motivation*"[MeSH Terms]) AND ("digital health application*"[Title/Abstract] OR "digital health technology*"[Title/Abstract] OR "digital health implementation*"[Title/Abstract] OR "digital health intervention*"[Title/Abstract] OR "digital instrument*"[Title/Abstract] OR "eHealth"[Title/Abstract] OR "social media*"[Title/Abstract] OR "social media*"[MeSH Terms] OR "wearable electronic device*"[Title/Abstract] OR "wearable electronic device*"[MeSH Terms] OR "reminder system*"[Title/Abstract] OR "reminder system*"[MeSH Terms] OR "mobile application*"[Title/Abstract] OR "mobile application*"[MeSH Terms] OR "cell phone*"[Title/Abstract] OR "cell phone*"[MeSH Terms] OR "mobile game based"[Title/Abstract] OR "game based tool*"[Title/Abstract] OR "gamification"[Title/Abstract] OR "serious game*"[Title/Abstract] OR "video game*"[Title/Abstract] OR "video game*"[MeSH Terms])<br>**Filters: Clinical Trial, Meta-Analysis, Randomized Controlled Trial, Review, Systematic Review, in the last 10 years**	329

#51	("adolescent*"[Title/Abstract] OR "adolescent*"[MeSH Terms] AND "diabetes mellitus"[Title/Abstract] OR "diabetes mellitus"[MeSH Terms]) AND ("health literacy"[Title/Abstract] OR "self management"[Title/Abstract] OR "self management"[MeSH Terms] OR "disease self management"[Title/Abstract] OR "self management of diabetes"[Title/Abstract] OR "self management education*"[Title/Abstract] OR "patient participation*"[Title/Abstract] OR "patient participation*"[MeSH Terms] OR "health education*"[Title/Abstract] OR "health behavior*"[Title/Abstract] OR "health behavior*"[MeSH Terms] OR "health knowledge*"[Title/Abstract] OR „motivation*"[Title/Abstract] OR „motivation*"[MeSH Terms]) AND ("digital health application*"[Title/Abstract] OR "digital health technology*"[Title/Abstract] OR "digital health implementation*"[Title/Abstract] OR "digital health intervention*"[Title/Abstract] OR "digital instrument*"[Title/Abstract] OR "eHealth"[Title/Abstract] OR "social media*"[Title/Abstract] OR "social media*"[MeSH Terms] OR "wearable electronic device*"[Title/Abstract] OR "wearable electronic device*"[MeSH Terms] OR "reminder system*"[Title/Abstract] OR "reminder system*"[MeSH Terms] OR "mobile application*"[Title/Abstract] OR "mobile application*"[MeSH Terms] OR "cell phone*"[Title/Abstract] OR "cell phone*"[MeSH Terms] OR "mobile game based"[Title/Abstract] OR "game based tool*"[Title/Abstract] OR "gamification"[Title/Abstract] OR "serious game*"[Title/Abstract] OR "video game*"[Title/Abstract] OR "video game*"[MeSH Terms])<br>**Filters: Clinical Trial, Meta-Analysis, Randomized Controlled Trial, Review, in the last 10 years**	311
#50	("adolescent*"[Title/Abstract] OR "adolescent*"[MeSH Terms] AND "diabetes mellitus"[Title/Abstract] OR "diabetes mellitus"[MeSH Terms]) AND ("health literacy"[Title/Abstract] OR "self management"[Title/Abstract] OR "self management"[MeSH Terms] OR "disease self management"[Title/Abstract] OR "self management of diabetes"[Title/Abstract] OR "self management education*"[Title/Abstract] OR "patient participation*"[Title/Abstract] OR "patient participation*"[MeSH Terms] OR "health education*"[Title/Abstract] OR "health behavior*"[Title/Abstract] OR "health behavior*"[MeSH Terms] OR "health knowledge*"[Title/Abstract] OR „motivation*"[Title/Abstract] OR „motivation*"[MeSH Terms]) AND ("digital health application*"[Title/Abstract] OR "digital health technology*"[Title/Abstract] OR "digital health implementation*"[Title/Abstract] OR "digital health intervention*"[Title/Abstract] OR "digital instrument*"[Title/Abstract] OR "eHealth"[Title/Abstract] OR "social media*"[Title/Abstract] OR "social media*"[MeSH Terms] OR "wearable electronic device*"[Title/Abstract] OR "wearable electronic device*"[MeSH Terms] OR "reminder system*"[Title/Abstract] OR "reminder system*"[MeSH Terms] OR "mobile application*"[Title/Abstract] OR "mobile application*"[MeSH Terms] OR "cell phone*"[Title/Abstract] OR "cell phone*"[MeSH Terms] OR "mobile game based"[Title/Abstract] OR "game based tool*"[Title/Abstract] OR "gamification"[Title/Abstract] OR "serious game*"[Title/Abstract] OR "video game*"[Title/Abstract] OR "video game*"[MeSH Terms])<br>**Filters: Clinical Trial, Meta-Analysis, Randomized Controlled Trial, in the last 10 years**	214

#49	("adolescent*"[Title/Abstract] OR "adolescent*"[MeSH Terms] AND "diabetes mellitus"[Title/Abstract] OR "diabetes mellitus"[MeSH Terms]) AND ("health literacy"[Title/Abstract] OR "self management"[Title/Abstract] OR "self management"[MeSH Terms] OR "disease self management"[Title/Abstract] OR "self management of diabetes"[Title/Abstract] OR "self management education*"[Title/Abstract] OR "patient participation*"[Title/Abstract] OR "patient participation*"[MeSH Terms] OR "health education*"[Title/Abstract] OR "health behavior*"[Title/Abstract] OR "health behavior*"[MeSH Terms] OR "health knowledge*"[Title/Abstract] OR „motivation*"[Title/Abstract] OR „motivation*"[MeSH Terms]) AND ("digital health application*"[Title/Abstract] OR "digital health technology*"[Title/Abstract] OR "digital health implementation*"[Title/Abstract] OR "digital health intervention*"[Title/Abstract] OR "digital instrument*"[Title/Abstract] OR "eHealth"[Title/Abstract] OR "social media*"[Title/Abstract] OR "social media*"[MeSH Terms] OR "wearable electronic device*"[Title/Abstract] OR "wearable electronic device*"[MeSH Terms] OR "reminder system*"[Title/Abstract] OR "reminder system*"[MeSH Terms] OR "mobile application*"[Title/Abstract] OR "mobile application*"[MeSH Terms] OR "cell phone*"[Title/Abstract] OR "cell phone*"[MeSH Terms] OR "mobile game based"[Title/Abstract] OR "game based tool*"[Title/Abstract] OR "gamification"[Title/Abstract] OR "serious game*"[Title/Abstract] OR "video game*"[Title/Abstract] OR "video game*"[MeSH Terms])<br>**Filters: Clinical Trial, Meta-Analysis, in the last 10 years**	214
#48	("adolescent*"[Title/Abstract] OR "adolescent*"[MeSH Terms] AND "diabetes mellitus"[Title/Abstract] OR "diabetes mellitus"[MeSH Terms]) AND ("health literacy"[Title/Abstract] OR "self management"[Title/Abstract] OR "self management"[MeSH Terms] OR "disease self management"[Title/Abstract] OR "self management of diabetes"[Title/Abstract] OR "self management education*"[Title/Abstract] OR "patient participation*"[Title/Abstract] OR "patient participation*"[MeSH Terms] OR "health education*"[Title/Abstract] OR "health behavior*"[Title/Abstract] OR "health behavior*"[MeSH Terms] OR "health knowledge*"[Title/Abstract] OR „motivation*"[Title/Abstract] OR „motivation*"[MeSH Terms]) AND ("digital health application*"[Title/Abstract] OR "digital health technology*"[Title/Abstract] OR "digital health implementation*"[Title/Abstract] OR "digital health intervention*"[Title/Abstract] OR "digital instrument*"[Title/Abstract] OR "eHealth"[Title/Abstract] OR "social media*"[Title/Abstract] OR "social media*"[MeSH Terms] OR "wearable electronic device*"[Title/Abstract] OR "wearable electronic device*"[MeSH Terms] OR "reminder system*"[Title/Abstract] OR "reminder system*"[MeSH Terms] OR "mobile application*"[Title/Abstract] OR "mobile application*"[MeSH Terms] OR "cell phone*"[Title/Abstract] OR "cell phone*"[MeSH Terms] OR "mobile game based"[Title/Abstract] OR "game based tool*"[Title/Abstract] OR "gamification"[Title/Abstract] OR "serious game*"[Title/Abstract] OR "video game*"[Title/Abstract] OR "video game*"[MeSH Terms])<br>**Filters: Clinical Trial, in the last 10 years**	182

#47	("adolescent*"[Title/Abstract] OR "adolescent*"[MeSH Terms] AND "diabetes mellitus"[Title/Abstract] OR "diabetes mellitus"[MeSH Terms]) AND ("health literacy"[Title/Abstract] OR "self management"[Title/Abstract] OR "self management"[MeSH Terms] OR "disease self management"[Title/Abstract] OR "self management of diabetes"[Title/Abstract] OR "self management education*"[Title/Abstract] OR "patient participation*"[Title/Abstract] OR "patient participation*"[MeSH Terms] OR "health education*"[Title/Abstract] OR "health behavior*"[Title/Abstract] OR "health behavior*"[MeSH Terms] OR "health knowledge*"[Title/Abstract] OR „motivation*"[Title/Abstract] OR „motivation*"[MeSH Terms]) AND ("digital health application*"[Title/Abstract] OR "digital health technology*"[Title/Abstract] OR "digital health implementation*"[Title/Abstract] OR "digital health intervention*"[Title/Abstract] OR "digital instrument*"[Title/Abstract] OR "eHealth"[Title/Abstract] OR "social media*"[Title/Abstract] OR "social media*"[MeSH Terms] OR "wearable electronic device*"[Title/Abstract] OR "wearable electronic device*"[MeSH Terms] OR "reminder system*"[Title/Abstract] OR "reminder system*"[MeSH Terms] OR "mobile application*"[Title/Abstract] OR "mobile application*"[MeSH Terms] OR "cell phone*"[Title/Abstract] OR "cell phone*"[MeSH Terms] OR "mobile game based"[Title/Abstract] OR "game based tool*"[Title/Abstract] OR "gamification"[Title/Abstract] OR "serious game*"[Title/Abstract] OR "video game*"[Title/Abstract] OR "video game*"[MeSH Terms])<br>**Filters: in the last 10 years**	682
#46	("adolescent*"[Title/Abstract] OR "adolescent*"[MeSH Terms] AND "diabetes mellitus"[Title/Abstract] OR "diabetes mellitus"[MeSH Terms]) AND ("health literacy"[Title/Abstract] OR "self management"[Title/Abstract] OR "self management"[MeSH Terms] OR "disease self management"[Title/Abstract] OR "self management of diabetes"[Title/Abstract] OR "self management education*"[Title/Abstract] OR "patient participation*"[Title/Abstract] OR "patient participation*"[MeSH Terms] OR "health education*"[Title/Abstract] OR "health behavior*"[Title/Abstract] OR "health behavior*"[MeSH Terms] OR "health knowledge*"[Title/Abstract] OR „motivation*"[Title/Abstract] OR „motivation*"[MeSH Terms]) AND ("digital health application*"[Title/Abstract] OR "digital health technology*"[Title/Abstract] OR "digital health implementation*"[Title/Abstract] OR "digital health intervention*"[Title/Abstract] OR "digital instrument*"[Title/Abstract] OR "eHealth"[Title/Abstract] OR "social media*"[Title/Abstract] OR "social media*"[MeSH Terms] OR "wearable electronic device*"[Title/Abstract] OR "wearable electronic device*"[MeSH Terms] OR "reminder system*"[Title/Abstract] OR "reminder system*"[MeSH Terms] OR "mobile application*"[Title/Abstract] OR "mobile application*"[MeSH Terms] OR "cell phone*"[Title/Abstract] OR "cell phone*"[MeSH Terms] OR "mobile game based"[Title/Abstract] OR "game based tool*"[Title/Abstract] OR "gamification"[Title/Abstract] OR "serious game*"[Title/Abstract] OR "video game*"[Title/Abstract] OR "video game*"[MeSH Terms])	780

#45	(((("adolescent*"[Title/Abstract]) OR ("adolescent*"[MeSH Terms])) AND (("diabetes mellitus"[Title/Abstract]) OR ("diabetes mellitus"[MeSH Terms]))) AND ((((((((((((((("health literacy"[Title/Abstract]) OR ("self management"[Title/Abstract])) OR ("self management"[MeSH Terms])) OR ("disease self management"[Title/Abstract])) OR ("self management of diabetes"[Title/Abstract])) OR ("self management education*"[Title/Abstract])) OR ("patient participation*"[Title/Abstract])) OR ("patient participation*"[MeSH Terms])) OR ("health education*"[Title/Abstract])) OR ("health behavior*"[Title/Abstract])) OR ("health behavior*"[MeSH Terms])) OR ("health knowledge*"[Title/Abstract])) OR ("motivation*"[Title/Abstract])) OR ("motivation*"[MeSH Terms])))) AND (((((((((((((((((((((("digital health application*"[Title/Abstract]) OR ("digital health technology*"[Title/Abstract])) OR ("digital health implementation*"[Title/Abstract])) OR ("digital health intervention*"[Title/Abstract])) OR ("digital instrument*"[Title/Abstract])) OR ("eHealth"[Title/Abstract])) OR ("social media*"[Title/Abstract])) OR ("social media*"[MeSH Terms])) OR ("wearable electronic device*"[Title/Abstract])) OR ("wearable electronic device*"[MeSH Terms])) OR ("reminder system*"[Title/Abstract])) OR ("reminder system*"[MeSH Terms])) OR ("mobile application*"[Title/Abstract])) OR ("mobile application*"[MeSH Terms])) OR ("cell phone*"[Title/Abstract])) OR ("cell phone*"[MeSH Terms])) OR ("mobile game based"[Title/Abstract])) OR ("game based tool*"[Title/Abstract])) OR ("gamification"[Title/Abstract])) OR ("serious game*"[Title/Abstract])) OR ("video game*"[Title/Abstract])) OR ("video game*"[MeSH Terms]))	146
#44	((((((((((((((((((((("digital health application*"[Title/Abstract]) OR ("digital health technology*"[Title/Abstract])) OR ("digital health implementation*"[Title/Abstract])) OR ("digital health intervention*"[Title/Abstract])) OR ("digital instrument*"[Title/Abstract])) OR ("eHealth"[Title/Abstract])) OR ("social media*"[Title/Abstract])) OR ("social media*"[MeSH Terms])) OR ("wearable electronic device*"[Title/Abstract])) OR ("wearable electronic device*"[MeSH Terms])) OR ("reminder system*"[Title/Abstract])) OR ("reminder system*"[MeSH Terms])) OR ("mobile application*"[Title/Abstract])) OR ("mobile application*"[MeSH Terms])) OR ("cell phone*"[Title/Abstract])) OR ("cell phone*"[MeSH Terms])) OR ("mobile game based"[Title/Abstract])) OR ("game based tool*"[Title/Abstract])) OR ("gamification"[Title/Abstract])) OR ("serious game*"[Title/Abstract])) OR ("video game*"[Title/Abstract])) OR ("video game*"[MeSH Terms])	82,667
#43	(((((((((((((("health literacy"[Title/Abstract]) OR ("self management"[Title/Abstract])) OR ("self management"[MeSH Terms])) OR ("disease self management"[Title/Abstract])) OR ("self management of diabetes"[Title/Abstract])) OR ("self management education*"[Title/Abstract])) OR ("patient participation*"[Title/Abstract])) OR ("patient participation*"[MeSH Terms])) OR ("health education*"[Title/Abstract])) OR ("health behavior*"[Title/Abstract])) OR ("health behavior*"[MeSH Terms])) OR ("health knowledge*"[Title/Abstract])) OR ("motivation*"[Title/Abstract])) OR ("motivation*"[MeSH Terms]))	639,470
#42	("diabetes mellitus"[Title/Abstract]) OR ("diabetes mellitus"[MeSH Terms])	537,810
#41	("adolescent*"[Title/Abstract]) OR ("adolescent*"[MeSH Terms])	2,196,135
#40	"video game*"[MeSH Terms]	6,276
#39	"video game*"[Title/Abstract]	4,016

#38	"serious game*"[Title/Abstract]	972
#37	"gamification"[Title/Abstract]	864
#36	"game based tool*"[Title/Abstract]	5
#35	"mobile game based"[Title/Abstract]	10
#34	"cell phone*"[MeSH Terms]	18,720
#33	"cell phone*"[Title/Abstract]	3,040
#32	"mobile application*"[MeSH Terms]	8,690
#31	"mobile application*"[Title/Abstract]	4,090
#30	"reminder system*"[MeSH Terms]	3,678
#29	"reminder system*"[Title/Abstract]	908
#28	"wearable electronic device*"[MeSH Terms]	14,829
#27	"wearable electronic device*"[Title/Abstract]	453
#26	"social media*"[MeSH Terms]	11,363
#25	"social media*"[Title/Abstract]	18,405
#24	"eHealth"[Title/Abstract]	7,340
#23	"digital instrument*"[Title/Abstract]	88
#22	"digital health intervention*"[Title/Abstract]	399
#21	"digital health implementation*"[Title/Abstract]	11
#20	"digital health technology*"[Title/Abstract]	151
#19	"digital health application*"[Title/Abstract]	72

#18	"motivation*"[MeSH Terms]	182,133
#17	"motivation*"[Title/Abstract]	98,917
#16	"health knowledge*"[Title/Abstract]	3,954
#15	"health behavior*"[MeSH Terms]	342,015
#14	"health behavior*"[Title/Abstract]	20,450
#13	"health education*"[Title/Abstract]	38,382
#12	"patient participation*"[MeSH Terms]	27,722
#11	"patient participation*"[Title/Abstract]	3,230
#10	"self management education*"[Title/Abstract]	1,554
#9	"self management of diabetes"[Title/Abstract]	301
#8	"disease self management"[Title/Abstract]	943
#7	"self management"[MeSH Terms]	3,712
#6	"self management"[Title/Abstract]	22,345
#5	"health literacy"[Title/Abstract]	10,862
#4	"diabetes mellitus"[MeSH Terms]	456,057
#3	"diabetes mellitus"[Title/Abstract]	228,016
#2	"adolescent*"[MeSH Terms]	2,126,787
#1	"adolescent*"[Title/Abstract]	285,319

The Cochrane Library_Date Run: 05/10/2021 09:18:27

ID	Search	Hits
#1	MeSH descriptor: [Diabetes Mellitus, Type 1] explode all trees	5792
#2	MeSH descriptor: [Diabetes Mellitus, Type 2] explode all trees	18904
#3	Diabetes Mellitus, Type 1:ti,ab	43975
#4	Diabetes Mellitus, Type 2:ti,ab	51933
#5	MeSH descriptor: [Adolescent] explode all trees	107314
#6	Adolescent*:ti,ab	28485
#7	#1 OR #2 OR # 3 OR #4	1042416
#8	#5 OR #6	126422
#9	#7 AND #8	73923
#10	MeSH descriptor: [Digital Technology] explode all trees	5
#11	"Digital Technology*":ti,ab	123
#12	MeSH descriptor: [Digital Divide] explode all trees	2
#13	"Digital Devide":ti,ab	0
#14	MeSH descriptor: [Telemedicine] explode all trees	2968
#15	Telemedicine:ti,ab	1813
#16	MeSH descriptor: [Social Media] explode all trees	208
#17	"Social Media":ti,ab	1485
#18	MeSH descriptor: [Wearable Electronic Devices] explode all trees	521

#19	"Wearable Electronic Device*":ti,ab	3
#20	MeSH descriptor: [Reminder Systems] explode all trees	988
#21	"Reminder System*":ti,ab	241
#22	MeSH descriptor: [Mobile Applications] explode all trees	909
#23	"Mobile Application*":ti,ab	1041
#24	MeSH descriptor: [Cell Phone] explode all trees	2021
#25	"Cell Phone*":ti,ab	586
#26	MeSH descriptor: [Video Games] explode all trees	759
#27	"Video Game*":ti,ab	761
#28	#10 OR #11 OR #12 OR #13 OR #14 OR #15 OR #16 OR #17 OR #18 OR #19 OR #20 OR #21 OR #22 OR #23 # OR #24 OR #25 #26 OR #27	10924
#29	#9 AND #28	965
#30	MeSH descriptor: [Health Literacy] explode all trees	417
#31	"Health literacy":ti,ab	1657
#32	MeSH descriptor: [Self-Management] explode all trees	567
#33	"Self-Management":ti,ab	8369
#34	MeSH descriptor: [Patient Education as Topic] explode all trees	9161
#35	"Patient Education":ti,ab	3476
#36	MeSH descriptor: [Health Education] explode all trees	20784
#37	"Health Education":ti,ab	5507
#38	MeSH descriptor: [Health Behavior] explode all trees	37142

#39	"Health Behavior*":ti,ab	2023
#40	MeSH descriptor: [Health Knowledge, Attitudes, Practice] explode all trees	6180
#41	"Health Knowledge":ti,ab	580
#42	MeSH descriptor: [Motivation] explode all trees	8768
#43	"Motivation*":ti,ab	8816
#44	#30 OR #31 OR #32 OR #33 OR #34 OR #35 OR #36 OR #37 OR #38 OR #39 OR #40 OR #41 OR #42 OR #43	79499
#45	#29 AND #44	474
#46	with Cochrane Library publication date from Jan 2011 to Oct 2021	419

Publikationstyp	Treffer
Cochrane Reviews	8
Cochrane Protocols	0
Trials	411
Editorials	0
Special collections	0
Clinical Answers	0

Embase_Date Run: 07/10/2021 15:02:21

No.	Query	Results
#48	#3 AND #6 AND #23 AND #46 AND [2011-2021]/py	106
#47	#3 AND #6 AND #23 AND #46	119

#46	#24 OR #25 OR #26 OR #27 OR #28 OR #29 OR #30 OR #31 OR #32 OR #33 OR #34 OR #35 OR #36 OR #37 OR #38 OR #39 OR #40 OR #41 OR #42 OR #43 OR #44 OR #45	108285
#45	'video game':ti,ab AND [embase]/lim	1953
#44	'video game'/exp AND [embase]/lim	2775
#43	'serious game':ti,ab AND [embase]/lim	317
#42	'serious game'/exp AND [embase]/lim	23
#41	'gamification':ti,ab AND [embase]/lim	523
#40	'gamification'/exp AND [embase]/lim	24
#39	'mobile phone':ti,ab AND [embase]/lim	6301
#38	'mobile phone'/exp AND [embase]/lim	27494
#37	'mobile application':ti,ab AND [embase]/lim	2447
#36	'mobile application'/exp AND [embase]/lim	12914
#35	'reminder system':ti,ab AND [embase]/lim	488
#34	'reminder system'/exp AND [embase]/lim	1286
#33	'wearable computer':ti,ab AND [embase]/lim	32
#32	'wearable computer'/exp AND [embase]/lim	4884
#31	'social media':ti,ab AND [embase]/lim	16248
#30	'social media'/exp AND [embase]/lim	23390
#29	'telehealth':ti,ab AND [embase]/lim	5711
#28	'telehealth'/exp AND [embase]/lim	42618
#27	'digital health technology':ti,ab AND [embase]/lim	135

#26	'digital health technology'/exp AND [embase]/lim	30
#25	'digital health intervention':ti,ab AND [embase]/lim	98
#24	'digital health intervention'/exp AND [embase]/lim	23
#23	#7 OR #8 OR #9 OR #10 OR #11 OR #12 OR #13 OR #14 OR #15 OR #16 OR #17 OR #18 OR #19 OR #20 OR #21 OR #22	277363
#22	'motivation':ti,ab AND [embase]/lim	54619
#21	'motivation'/exp AND [embase]/lim	83636
#20	'attitude to health':ti,ab AND [embase]/lim	53
#19	'attitude to health'/exp AND [embase]/lim	24958
#18	'diabetes education':ti,ab AND [embase]/lim	2998
#17	'diabetes education'/exp AND [embase]/lim	3744
#16	'patient education':ti,ab AND [embase]/lim	22180
#15	'patient education'/exp AND [embase]/lim	66673
#14	'patient participation':ti,ab AND [embase]/lim	2327
#13	'patient participation'/exp AND [embase]/lim	16613
#12	'diabetes self management education':ti,ab AND [embase]/lim	694
#11	'diabetes self management education'/exp AND [embase]/lim	31
#10	'self care':ti,ab AND [embase]/lim	19369
#9	'self care'/exp AND [embase]/lim	60153
#8	'health literacy':ti,ab AND [embase]/lim	8745
#7	'health literacy'/exp AND [embase]/lim	10183

#6	#4 OR #5	981388
#5	'diabetes mellitus':ti,ab AND [embase]/lim	263237
#4	'diabetes mellitus'/exp AND [embase]/lim	955969
#3	#1 OR #2	1049827
#2	'adolescent':ti,ab AND [embase]/lim	131199
#1	'adolescent'/exp AND [embase]/lim	1020035

Web of Science_ Date Run: 13/10/2021 14:21:58

#	Query	Results
41	#1 AND #2 AND #17 AND #39 and 2011 or 2012 or 2013 or 2014 or 2015 or 2016 or 2017 or 2018 or 2019 or 2020 or 2021 (Publica tion Years)	28
40	#1 AND #2 AND #17 AND #39	32
39	#18 OR #19 OR #19 OR #20 OR #21 OR #22 OR #23 OR #24 OR #24 OR #25 OR #26 OR #27 OR #28 OR #29 OR #30 OR #31 OR #32 OR #33 OR #34 OR #35 OR #36 OR #37 OR #38	147,741
38	TS="patient* empowerment"	1,745
37	TS="digital health apps"	16
36	TS="mobile apps"	3,040
35	TS="mobile phone*"	24,207
34	TS="video game*"	11,958
34	TS=telemedicine	22,660
32	TS="serious game*"	2,996

31	TS="gamification"	3,641
30	TS="games based tool*"	16
29	TS="mobile game based"	47
28	TS="cell phone*"	6,698
27	TS="reminder system*"	884
26	TS="mobile application*"	10,693
25	TS="wearable electronic device*"	1,107
24	TS="social media"	61,406
23	TS=ehealth	5,444
22	TS="digital instrument*"	501
21	TS="digital health intervention*"	413
20	TS="digital health implementation"	11
19	TS="digital health technology*"	146
18	TS="digital health application*"	91
17	#3 OR #4 OR #5 OR #6 OR #7 OR #8 OR #9 OR #10 OR #11 OR #12 OR #13 OR #14 OR #15 OR #16	306,743
16	TS="self-care"	22,379
15	TS="diabetes self management education"	735
14	TS="diabetes education"	2,696
13	TS="attitude to health"	422
12	TS="motivation"	178,584

11	TS="health knowledge"	4,628
10	TS="health behavior?"	14,421
9	TS="health education"	31,037
8	TS="patient* education"	20,498
7	TS="patient* participation"	4,011
6	TS="Self management education"	1,990
5	TS="Self management of diabetes"	272
4	TS=self-management	31,613
3	TS="health literacy"	14,274
2	TS=adolescent*	461,895
1	TS="diabetes mellitus"	268,693

PsycINFO_ Date Run: 12/10/2021 12:38:01

#	Query	Results
S31	S1 AND S2 AND S13 AND S29	26
S30	S1 AND S2 AND S13 AND S29	30
S29	S14 OR S15 OR S16 OR S17 OR S18 OR S19 OR S20 OR S21 OR S22 OR S23 OR S24 OR S25 OR S26 OR S27 OR S28	54,800
S28	gamification OR gamification OR gamification	819
S27	serious games OR serious games OR serious games	1,087
S26	game based intervention OR game based intervention OR game based intervention	183

S25	(mobile games or mobile game) OR (mobile games or mobile game) OR (mobile games or mobile game)	460
S24	cell phones OR cell phones OR cell phones	5,890
S23	(mobile applications or apps or mobile apps) OR (mobile applications or apps or mobile apps) OR (mobile applications or apps or mobile apps)	10,083
S22	(mobile applications or apps or mobile apps) OR (mobile applications or apps or mobile apps) OR (mobile applications or apps or mobile apps)	10,083
S21	reminder system OR reminder system OR reminder system	691
S20	wearable electronic devices OR wearable electronic devices OR wearable electronic devices	392
S19	social media OR social media OR social media	30,805
S18	(ehealth or mhealth or digital health) OR (ehealth or mhealth or digital health) OR (ehealth or mhealth or digital health)	8,734
S17	digital health intervention OR digital health intervention OR digital health intervention	195
S16	digital health technology OR digital health technology OR digital health technology	225
S15	digital health apps OR digital health apps OR digital health apps	29
S14	digital health applications OR digital health applications OR digital health applications	29
S13	S3 OR S4 OR S5 OR S6 OR S7 OR S8 OR S9 OR S10 OR S11 OR S12	228,387
S12	MA motivation OR TI motivation OR AB motivation	127,337
S11	MA health knowledge OR TI health knowledge OR AB health knowledge	36,207
S10	MA health education OR TI health education OR AB health education	33,388
S9	MA patient education OR TI patient education OR AB patient education	18,740
S8	MA (patient participation or patient involvement or patient empowerment or patient engagement) OR TI (patient participation or patient involvement or patient empowerment or patient engagement) OR AB (patient participation or patient involvement or patient empowerment or patient engagement)	13,805
S7	MA self management education for diabetes OR TI self management education for diabetes OR AB self management education for diabetes	363
S6	MA self management education OR TI self management education OR AB self management education	920

S5	MA self management of diabetes OR TI self management of diabetes OR AB self management of diabetes	1,796
S4	MA (self management or self-management) OR TI (self management or self-management) OR AB (self management or self-management)	14,506
S3	MA health literacy OR TI health literacy OR AB health literacy	5,028
S2	MA adolescents OR TI adolescents OR AB adolescents	436,359
S1	MA diabetes mellitus OR TI diabetes mellitus OR AB diabetes mellitus	15,452

Appendix 2: Scoring information – Results of the critical appraisal using the RoB2 tool

Study	Ayar et al. (2021)		Henkemans et al. (2017)		Newton & Ashley (2013)	
Preliminary considerations:	Study design: Individually Randomized Group-Treatment (IRGT) Trials<br>Intervention:<br>E: Web-based diabetes education (powerpoint, quizzes and blogs)<br>C: Diabetes education in clinical setting (tools: pamphlets and/or posters)<br>Outcome assessed for risk of bias:<br>1. Metabolic control<br>2. Self-efficacy<br>3. QoL<br>Numerical result being assessed: Metabolic control A1C: no significant difference between E and C (P > 0.05)<br>Effect of interest: intention-to-treat effect<br>Sources: Journal article with results of the trial		Study design: Individually Randomized Group-Treatment (IRGT) Trials<br>Intervention:<br>2 groups:<br>the personal robot, neutral robot or control group (care as usual)<br>Outcome assessed for risk of bias:<br>1. "Learning by playing with a robot”<br>2. Effects of personalisation on child-robot interaction in a clinical setting.<br>Numerical result being assessed: Knowledge: Results increase in E1 and E2 but not in C. (P = .001); SDT: higher score in E1 than E2 (P = .02); Pleasurable (P = .04), more questions correctly answered (P = .02), more motivation to play a fourth time (P =.03) with the robots.<br>More engagement, attention, more social and positive with E1 (P < .05).<br>Effect of interest: intention-to-treat effect<br>Sources: Journal article with results of the trial		Study design: Individually Randomized Group-Treatment (IRGT) Trials<br>Interventions: E = 7 weeks web-based intervention, taking part in problem-solving through discussion forums, chat rooms and blogs; C = standard medical care as usual.<br>Outcome assessed for risk of bias:<br>1. QoL for Youths; 2. Self-efficacy; 3. Outcome expectations<br>Numerical result being assessed: QoL: no significant differences between E and C (P = 0.63)<br>Self-efficacy: no significant differences between E and C (P = 0.53)<br>Negative Outcome Expectations (P = 0.31)<br>Effect of interest: intention-to-treat effect<br>Sources: Journal article with results of the trial	
Major Components	Response options	Evidence/quote/explanation	Response options	Evidence/quote/explanation	Response options	Evidence/quote/explanation
**Domain 1: Risk of bias arising from the randomization process**						
1.1 Was the allocation sequence random?	NI	The only information about randomization methods is a statement that the study is randomized.	NI	The children were allocated to the personal robot, neutral robot or control group (care as usual).	Y	SPSS software was used to randomize the participants to control or experimental group.

Study	Ayar et al. (2021)		Henkemans et al. (2017)		Newton & Ashley (2013)	
1.2 Was the allocation sequence concealed until participants were enrolled and assigned to interventions?	Y	The first patient was allocated to the control group and the second patient to the intervention group and so on.	NI	„Parents and children received a letter with information about the study (goal, results, contribution to ALIZ-e project, data processing and rights) and an invitation to participate in the study. Parents gave written informed consent for participation of their children in the study and the children provided verbal assent and an initialled consent form.“ But NI if participant had knowledge of the forthcoming allocation.	PY	
1.3 Did baseline differences between intervention groups suggest a problem with the randomization process?	N	„Analyses indicated that the experimental and control groups did not differ in terms of age, sex, diabetes history, initial A1C levels, and initial mean scores of self-management and self-efficacy in the diabetes scale for the adolescents with type 1 diabetes and the QOL in children with diabetes scale.” (Aya 2021: 135)	NI		PN	
Risk-of-bias judgement	Low		Some concerns		Low	
Optional: what is the predicted direction of bias arising from the randomisation process?	NA		NA		NA	
**Domain 2: Risk of bias due to deviations from the intended interventions (effect of assignment to intervention)**						
2.1. Were participants aware of their assigned intervention during the trial?	NI		PY	They received all the informations.	NI	
2.2. Were carers and people delivering the interventions aware of participants' assigned intervention during the trial?	PY	Not explicit but interaction HCP – T1DM	PY	Not explicit but interaction HCP – T1DM	PY	
2.3. If Y/PY/NI to 2.1 or 2.2: Were there deviations from the intended intervention that arose because of the trial context?	**PY**	four of the adolescents' use of internet was limited at home, and two adolescents did not use the site actively. The researchers could not contact two control group adolescents at the end of the third month.	**NI**	Trialists do not always report whether deviations arose because of the trial context.	**PY**	There was a general decline in the number and duration of web site visits from the beginning to the end of the study
2.4 If Y/PY to 2.3: Were these deviations likely to have affected the outcome?	PY		NA		PN	
2.5. If Y/PY/NI to 2.4: Were these deviations from intended intervention balanced between groups?	**PY**		**NA**			
2.6 Was an appropriate analysis used to estimate the effect of assignment to intervention?	PY	5.7% (4 individuals)	NI		NI	

Study	Ayar et al. (2021)		Henkemans et al. (2017)		Newton & Ashley (2013)	
2.7 If N/PN/NI to 2.6: Was there potential for a substantial impact (on the result) of the failure to analyse participants in the group to which they were randomized?	NA		PN		PN	
Optional: What is the predicated direction of bias due to deviations from intended interventions?	NA		NA		NA	
Risk-of-bias judgement	Part 1: some concerns, Part 2: Low risk<br>Some concerns		Part 1: some concerns, Part 2: Some concerns<br>Some concerns		Part 1: some concerns, Part 2: Some concerns<br>Some concerns	
**Domain 2: Risk of bias due to deviations from the intended interventions (effect of adhering to intervention)**						
2.1. Were participants aware of their assigned intervention during the trial?						
2.2. Were carers and people delivering the interventions aware of participants' assigned intervention during the trial?						
2.3. [If applicable:] If Y/PY/NI to 2.1 or 2.2: Were important non- protocol interventions balanced across intervention groups?						
2.4. [If applicable:] Were there failures in implementing the intervention that could have affected the outcome?						
2.5. [If applicable:] Was there non-adherence to the assigned intervention regimen that could have affected participants' outcomes?						
2.6. If N/PN/NI to 2.3, or Y/PY/NI to 2.4 or 2.5: Was an appropriate analysis used to estimate the effect of adhering to the intervention?						
**Domain 3: Risk of bias due to missing outcome data**						
3.1 Were data for this outcome available for all, or nearly all, participants randomized?	Y	Dropout rate 5.7%: 4 individual	Y	Dropout: 1 individual	Y	
3.2 If N/PN/NI to 3.1: Is there evidence that the result was not biased by missing outcome data?	NA		NA		NA	

Study	Ayar et al. (2021)		Henkemans et al. (2017)		Newton & Ashley (2013)	
3.3 If N/PN to 3.2: Could missingness in the outcome depend on its true value?	NA		NA		NA	
3.4 If Y/PY/NI to 3.3: Is it likely that missingness in the outcome depended on its true value?	NA		NA		NA	
Optional: what is the predicted direction of bias due to missing outcome data?	Towards null		Towards null		Towards null	
Risk-of-bias judgement	Low		Low		Low	
**Domain 4: Risk of bias in measurement of the outcome**						
4.1 Was the method of measuring the outcome inappropriate?	N		N		PN	
4.2 Could measurement or ascertainment of the outcome have differed between intervention groups?	PN		PN		NI	
4.3 If N/PN/NI to 4.1 and 4.2: Were outcome assessors aware of the intervention received by study participants?	PY		PY		PY	
4.4 If Y/PY/NI to 4.3: Could assessment of the outcome have been influenced by knowledge of intervention received?	NI		NI		NI	
4.5 If Y/PY/NI to 4.4: Is it likely that assessment of the outcome was influenced by knowledge of intervention received?	PN		PN		PN	
Optional: what is the predicated direction of bias in measurement of the outcome?	NA		NA		NA	
Risk-of-bias judgement	Some concerns		Some concerns		Some concerns	
**Domain 5: Risk of bias in selection of the reported result**						
5.1 Were the data that produced this result analysed in accordance with a pre-specified analysis plan that was finalized before unblinded outcome data were available for analysis?	NI		NI		NI	
Is the numerical result being assessed likely to have been selected, on the basis of the results, from...<br>5.2. ... multiple eligible outcome measurements (e.g. scales, definitions, time points) within the outcome domain?	NI		NI		NI	

Study	Ayar et al. (2021)		Henkemans et al. (2017)		Newton & Ashley (2013)	
5.3. ... multiple eligible analyses of the data?	NI	Analysis intentions are not available	NI	Analysis intentions are not available	NI	Analysis intentions are not available
Optional: what is the overall predicted direction ob bias for this outcome?	NA		NA		NA	
Risk-of-bias judgement	Some concerns		Some concerns		Some concerns	
**Overall risk-of-bias judgement**	Some concerns		Some concerns		Some concerns	
Optional: what is the overall predicted direction of bias for this outcome?	NA		NA		NA	

Appendix 3: Scoring information – Results of the critical appraisal using the ROBINS-I

Study	Sap et al. (2019)	
Preliminary considerations:	Participants: T1DM 13–26; Experimental intervention: patient education trought WhatsApp; Comparator: classic follow-up; Outcomes: knowledge and glycemic control<br>Effect of assignment to intervention	
Major Components	Response options	Evidence/quote/explanation
Domain 1: Bias due to confounding		
1.1 Is there potential for confounding of the effect of intervention in this study?	Y	adolescents and young having a smartphone with internet access<br>Limitation: „Android phones were not provided to patients, we recruited only those with their own phones: this might have caused selection bias“.<br>characteristics of the study population: They were all students.
If Y/PY to 1.1: determine whether there is a need to assess time-varying confounding:<br>1.2. Was the analysis based on splitting participants' follow up time according to intervention received?	PN	
1.3. Were intervention discontinuations or switches likely to be related to factors that are prognostic for the outcome?	-	(If Y/PY in question 1.2.)
1.4. Did the authors use an appropriate analysis method that controlled for all the important confounding domains?	N	
1.5. If Y/PY to 1.4: Were confounding domains that were controlled for measured validly and reliably by the variables available in this study?	-	

1.6. Did the authors control for any post-intervention variables that could have been affected by the intervention?	N	
1.7. Did the authors use an appropriate analysis method that adjusted for all the important confounding domains and for time- varying confounding?	-	
1.8. If Y/PY to 1.7: Were confounding domains that were adjusted for measured validly and reliably by the variables available in this study?	-	
Optional: What is the predicted direction of bias due to confounding?	-	
Risk of bias judgement	Serious risk of bias	(i) At least one known important domain was not appropriately measured, or not controlled for;<br>or<br>(ii) Reliability or validity of measurement of an important domain was low enough that we expect serious residual confounding.
Domain 2: Bias in selection of participants into the study		
2.1. Was selection of participants into the study (or into the analysis) based on participant characteristics observed after the start of intervention?<br>If N/PN to 2.1: go to 2.4	N	
2.2. If Y/PY to 2.1: Were the post-intervention variables that influenced selection likely to be associated with intervention?	-	
2.3 If Y/PY to 2.2: Were the post-intervention variables that influenced selection likely to be influenced by the outcome or a cause of the outcome?	-	
2.4. Do start of follow-up and start of intervention coincide for most participants?	NI	
2.5. If Y/PY to 2.2 and 2.3, or N/PN to 2.4: Were adjustment techniques used that are likely to correct for the presence of selection biases?	N	
Optional: What is the predicted direction of bias due to selection of participants into the study?	Towards null	
Risk of bias judgement	Moderate risk of bias	We don't know if „For each participant, start of follow up and start of intervention coincided".
Domain 3: Bias in classification of interventions		
3.1 Were intervention groups clearly defined?	Y	
3.2 Was the information used to define intervention groups recorded at the start of the intervention?	Y	

3.3 Could classification of intervention status have been affected by knowledge of the outcome or risk of the outcome?	NI	
Optional: What is the predicted direction of bias due to measurement of outcomes or interventions?	Towards null	
Risk of bias judgement	Low risk of bias	
Domain 4: Bias due to deviations from intended interventions		
If your aim for this study is to assess the effect of assignment to intervention, answer questions 4.1 and 4.2<br>4.1. Were there deviations from the intended intervention beyond what would be expected in usual practice?	NI	
4.2. If Y/PY to 4.1: Were these deviations from intended intervention unbalanced between groups and likely to have affected the outcome?	-	
If your aim for this study is to assess the effect of starting and adhering to intervention, answer questions 4.3 to 4.6<br>4.3. Were important co-interventions balanced across intervention groups?	-	
4.4. Was the intervention implemented successfully for most participants?	-	
4.5. Did study participants adhere to the assigned intervention regimen?		
4.6. If N/PN to 4.3, 4.4 or 4.5: Was an appropriate analysis used to estimate the effect of starting and adhering to the intervention?	-	
Optional: What is the predicted direction of bias due to deviations from the intended interventions?	Towards null	
Risk of bias judgement	No information	No information is reported on whether there is deviation from the intended intervention.
Domain 5: Bias due to missing data		
5.1 Were outcome data available for all, or nearly all, participants?	Y	
5.2 Were participants excluded due to missing data on intervention status?	NI	
5.3 Were participants excluded due to missing data on other variables needed for the analysis?	N	
5.4 If PN/N to 5.1, or Y/PY to 5.2 or 5.3: Are the proportion of participants and reasons for missing data similar across interventions?	-	

5.5 If PN/N to 5.1, or Y/PY to 5.2 or 5.3: Is there evidence that results were robust to the presence of missing data?	-	
Optional: What is the predicted direction of bias due to missing data?	Towards null	
Risk of bias judgement	Low risk of bias	
Domain 6: Bias in measurement of outcomes		
6.1 Could the outcome measure have been influenced by knowledge of the intervention received?	PN	
6.2 Were outcome assessors aware of the intervention received by study participants?	PN	
6.3 Were the methods of outcome assessment comparable across intervention groups?	Y	
6.4 Were any systematic errors in measurement of the outcome related to intervention received?	N	
Optional: What is the predicted direction of bias due to measurement of outcomes?	Towards null	
Risk of bias judgement	Risk of bias judgement	
Domain 7: Bias in selection of the reported result		
Is the reported effect estimate likely to be selected, on the basis of the results, from...<br>7.1. ... multiple outcome measurements within the outcome domain?	N	
7.2 ... multiple analyses of the intervention- outcome relationship?	NI	
7.3 ... different subgroups?	NI	
Optional: What is the predicted direction of bias due to selection of the reported result?	-	
Risk of bias judgement	No information	There is too little information to make a judgement
Overall risk-of-bias judgement	Serious risk of bias	„The study is judged to be at serious risk of bias in at least one domain, but not at critical risk of bias in any domain“
Optional: what is the overall predicted direction of bias for this outcome?	-	

Appendix 4: Scoring information – Results of the critical appraisal using the NOS

Study	Döğer et al. (2019)	
Study design	Cohort study	
Major Components	Response options	Description
Selection		
1) Representativeness of the exposed cohort	⁕	82 Children and Adolescents with Type 1 Diabetes but: „The limited number of cases constitute a definite limitation of the study“
2) Selection of the non exposed cohort	-	No description of the derivation of the non exposed cohort
3) Ascertainment of exposure	-	Written self report
4) Demonstration that outcome of interest was not present at start of study	⁕	Conflict of interest: None declared
Comparability		
1) Comparability of cases and controls on the basis of the design or analysis	-	No description
Outcome		
1) Assessment of outcome	-	No description
2) Was follow-up long enough for outcomes to occur	-	No: „The short duration of the study could be construed as a limitation although significant differences were detected and have been detected in shorter duration studies than this one „
3) Adequacy of follow up of cohorts		No description
Total score	2	

Appendix 5: Scoring information – Results of the critical appraisal using the CASP

Study	Pembroke et al. (2021)			Malik et al. (2019)			Troncone et al. (2019)			Vaala et al. (2018)			Frøisland & Årsand (2015)			Nordfeldt et al. (2013)		
Major Components	Response options																	
**Section A: Are the results valid?**																		
1. Was there a clear statement of the aims of the research?	**Yes**	No	Can't tell	**Yes**	No	Can't tell	**Yes**	No	Can't tell	**Yes**	No	Can't tell	**Yes**	No	Can't tell	**Yes**	No	Can't tell
2. Is a qualitative methodology appropriate?	**Yes**	No	Can't tell	**Yes**	No	Can't tell	Yes	No	**Can't tell**	Yes	No	**Can't tell**	**Yes**	No	Can't tell	**Yes**	No	Can't tell
3. Was the research design appropriate to address the aims of the research?	Yes	No	**Can't tell**	Yes	No	**Can't tell**	Yes	No	**Can't tell**	**Yes**	No	Can't tell	Yes	No	**Can't tell**	Yes	No	**Can't tell**
4. Was the recruitment strategy appropriate to the aims of the research?	**Yes**	No	Can't tell	**Yes**	No	Can't tell	**Yes**	No	Can't tell	**Yes**	No	Can't tell	**Yes**	No	Can't tell	**Yes**	No	Can't tell
5. Was the data collected in a way that addressed the research issue?	**Yes**	No	Can't tell	**Yes**	No	Can't tell	Yes	**No**	Can't tell	**Yes**	No	Can't tell	**Yes**	No	Can't tell	**Yes**	No	Can't tell
6. Has the relationship between researcher and participants been adequately considered?	Yes	No	**Can't tell**	Yes	No	**Can't tell**	Yes	No	**Can't tell**	Yes	**No**	Can't tell	Yes	No	**Can't tell**	Yes	**No**	Can't tell
**Section B: what are the results?**																		
7. Have ethical issues been taken into consideration?	**Yes**	No	Can't tell	**Yes**	No	Can't tell	**Yes**	No	Can't tell	Yes	No	**Can't tell**	**Yes**	No	Can't tell	**Yes**	No	Can't tell
8. Was the data analysis sufficiently rigorous?	**Yes**	No	Can't tell	**Yes**	No	Can't tell	**Yes**	No	Can't tell	**Yes**	No	Can't tell	**Yes**	No	Can't tell	**Yes**	No	Can't tell
9. Is there a clear statement of findings?	**Yes**	No	Can't tell	**Yes**	No	Can't tell	**Yes**	No	Can't tell	**Yes**	No	Can't tell	**Yes**	No	Can't tell	**Yes**	No	Can't tell
**Section C: Will the results help locally?**																		
10. How valuable is the research?	Valuable			Valuable			Valuable			Valuable			Valuable			Valuable		
TOTAL SCORE	7		2	7		2	5	1	3	6	1	2	7		2	7	1	1

Appendix 6: Scoring information – Results of the critical appraisal using the Amstar-2 tool

Study	Nkhoma et al. (2021)			Zaho et al. (2021)			Rewolinski et al. (2018)			Duke et al. (2018)			Chaves et al. (2017)		
Major Components	Response options														
1. Did the research questions and inclusion criteria for the review include the components of PICO?	**Yes**	No	/	**Yes**	No	/	**Yes**	No	/	**Yes**	No	/	**Yes**	No	/
2. Did the report of the review contain an explicit statement that the review methods were established prior to the conduct of the review and did the report justify any significant deviations from the protocol?	**Yes**	No	Partial Yes	Yes	**No**	Partial Yes	Yes	**No**	Partial Yes	**Yes**	No	Partial Yes	Yes	**No**	Partial Yes
3. Did the review authors explain their selection of the study designs for inclusion in the review?	**Yes**	No	/	**Yes**	No	/	**Yes**	No	/	**Yes**	No	/	**Yes**	No	/
4. Did the review authors use a comprehensive literature search strategy?	**Yes**	No	Partial Yes	**Yes**	No	Partial Yes	**Yes**	No	Partial Yes	**Yes**	No	Partial Yes	**Yes**	No	Partial Yes
5. Did the review authors perform study selection in duplicate?	**Yes**	No	/	**Yes**	No	/	Yes	**No**	/	Yes	**No**	/	**Yes**	No	/
6. Did the review authors perform data extraction in duplicate?	**Yes**	No	/	Yes	**No**	/	Yes	**No**	/	Yes	**No**	/	**Yes**	No	/
7. Did the review authors provide a list of excluded studies and justify the exclusions?	**Yes**	No	Partial Yes	Yes	No	**Partial Yes**	**Yes**	No	Partial Yes	Yes	**No**	Partial Yes	Yes	**No**	Partial Yes
8. Did the review authors describe the included studies in adequate detail?	**Yes**	No	Partial Yes	**Yes**	No	Partial Yes	**Yes**	No	Partial Yes	**Yes**	No	Partial Yes	**Yes**	No	Partial Yes
9. Did the review authors use a satisfactory technique for assessing the risk of bias (RoB) in individual studies that were included in the review?	**Yes**	No	Partial Yes	**Yes**	No	Partial Yes	**Yes**	No	Partial Yes	Yes	**No**	Partial Yes	Yes	**No**	Partial Yes

Study	Nkhoma et al. (2021)			Zaho et al. (2021)			Rewolinski et al. (2018)			Duke et al. (2018)			Chaves et al. (2017)		
10. Did the review authors report on the sources of funding for the studies included in the review?	**Yes**	No	/	**Yes**	No	/	**Yes**	No	/	**Yes**	No	/	**Yes**	No	/
11. If meta-analysis was performed did the review authors use appropriate methods for statistical combination of results?	**Yes**	No	No meta-analysis conducted	Yes	No	**No meta-analysis conducted**	Yes	No	**No meta-analysis conducted**	Yes	No	**No meta-analysis conducted**	Yes	No	**No meta-analysis conducted**
12. If meta-analysis was performed, did the review authors assess the potential impact of RoB in individual studies on the results of the meta-analysis or other evidence synthesis?	**Yes**	No	No meta-analysis conducted	Yes	No	**No meta-analysis conducted**	Yes	No	**No meta-analysis conducted**	Yes	No	**No meta-analysis conducted**	Yes	No	**No meta-analysis conducted**
13. Did the review authors account for RoB in individual studies when interpreting/ discussing the results of the review?	**Yes**	No	/	**Yes**	No	/	**Yes**	No	/	Yes	**No**	/	Yes	**No**	/
14. Did the review authors provide a satisfactory explanation for, and discussion of, any heterogeneity observed in the results of the review?	**Yes**	No	/	**Yes**	No	/	**Yes**	No	/	**Yes**	No	/	**Yes**	No	/
15. If they performed quantitative synthesis did the review authors carry out an adequate investigation of publication bias (small study bias) and discuss its likely impact on the results of the review?	**Yes**	No	No meta-analysis conducted	Yes	No	**No meta-analysis conducted**	Yes	No	**No meta-analysis conducted**	Yes	No	**No meta-analysis conducted**	Yes	No	**No meta-analysis conducted**
16. Did the review authors report any potential sources of conflict of interest, including any funding they received for conducting the review?	**Yes**	No	/	**Yes**	No	/	Yes	**No**	/	**Yes**	No	/	**Yes**	No	/
TOTAL SCORE (out of 16)	16			10	1	4	9			8	5		9	4	
Overall methodological quality (L = low, M = moderate, H = high)	High quality review			Low quality review			Low quality review			Critically Low quality review			Critically Low quality review		

Study	Swartwout et al. (2016)			Lazem et al. (2015)			McDarby et al. (2015)			Pal (2014)			Ho et al. (2014)			Dougherty et al. (2014)		
Major Components																		
1. Did the research questions and inclusion criteria for the review include the components of PICO?	Yes	**No**	/	**Yes**	No	/	Yes	**No**	/	Yes	**No**	/	Yes	**No**	/	**Yes**	No	/
2. Did the report of the review contain an explicit statement that the review methods were established prior to the conduct of the review and did the report justify any significant deviations from the protocol?	Yes	**No**	Partial Yes	Yes	**No**	Partial Yes	Yes	**No**	Partial Yes	Yes	**No**	Partial Yes	Yes	No	**Partial Yes**	Yes	**No**	Partial Yes
3. Did the review authors explain their selection of the study designs for inclusion in the review?	**Yes**	No	/	**Yes**	No	/	Yes	**No**	/	Yes	**No**	/	**Yes**	No	/	**Yes**	No	/
4. Did the review authors use a comprehensive literature search strategy?	**Yes**	No	Partial Yes	**Yes**	No	Partial Yes	Yes	**No**	Partial Yes	Yes	**No**	Partial Yes	Yes	No	**Partial Yes**	**Yes**	No	Partial Yes
5. Did the review authors perform study selection in duplicate?	Yes	**No**	/	**Yes**	No	/	Yes	**No**	/	Yes	**No**	/	**Yes**	No	/	Yes	**No**	/
6. Did the review authors perform data extraction in duplicate?	Yes	**No**	/	**Yes**	No	/	Yes	**No**	/	Yes	**No**	/	**Yes**	No	/	Yes	**No**	/
7. Did the review authors provide a list of excluded studies and justify the exclusions?	Yes	**No**	Partial Yes	Yes	No	**Partial Yes**	Yes	**No**	Partial Yes	Yes	**No**	Partial Yes	Yes	No	**Partial Yes**	Yes	No	**Partial Yes**
8. Did the review authors describe the included studies in adequate detail?	**Yes**	No	Partial Yes	Yes	**No**	Partial Yes	Yes	**No**	Partial Yes	Yes	**No**	Partial Yes	**Yes**	No	Partial Yes	Yes	No	**Partial Yes**
9. Did the review authors use a satisfactory technique for assessing the risk of bias (RoB) in individual studies that were included in the review?	Yes	**No**	Partial Yes	Yes	**No**	Partial Yes	Yes	**No**	Partial Yes	Yes	**No**	Partial Yes	Yes	**No**	Partial Yes	Yes	**No**	Partial Yes
10. Did the review authors report on the sources of funding for the studies included in the review?	Yes	**No**	/	Yes	**No**	/	Yes	**No**	/	Yes	**No**	/	**Yes**	No	/	**Yes**	No	/
11. If meta-analysis was performed did the review authors use appropriate methods for statistical combination of results?	Yes	No	**No meta-analysis conducted**	Yes	No	**No meta-analysis conducted**	Yes	No	**No meta-analysis conducted**	Yes	No	**No meta-analysis conducted**	Yes	No	**No meta-analysis conducted**	Yes	No	**No meta-analysis conducted**
12. If meta-analysis was performed, did the review authors assess the potential impact of RoB in individual studies on the results of the meta-analysis or other evidence synthesis?	Yes	No	**No meta-analysis conducted**	Yes	No	**No meta-analysis conducted**	Yes	No	**No meta-analysis conducted**	Yes	No	**No meta-analysis conducted**	Yes	No	**No meta-analysis conducted**	Yes	No	**No meta-analysis conducted**
13. Did the review authors account for RoB in individual studies when interpreting/ discussing the results of the review?	Yes	**No**	/	Yes	**No**	/	Yes	**No**	/	Yes	**No**	/	Yes	**No**	/	Yes	**No**	/
14. Did the review authors provide a satisfactory explanation for, and discussion of, any heterogeneity observed in the results of the review?	Yes	**No**	/	Yes	**No**	/	Yes	**No**	/	Yes	**No**	/	Yes	**No**	/	Yes	**No**	/

Study	Swartwout et al. (2016)			Lazem et al. (2015)			McDarby et al. (2015)			Pal (2014)			Ho et al. (2014)			Dougherty et al. (2014)		
15. If they performed quantitative synthesis did the review authors carry out an adequate investigation of publication bias (small study bias) and discuss its likely impact on the results of the review?	Yes	No	**No meta-analysis conducted**	Yes	No	**No meta-analysis conducted**	Yes	No	**No meta-analysis conducted**	Yes	No	**No meta-analysis conducted**	Yes	No	**No meta-analysis conducted**	Yes	No	**No meta-analysis conducted**
16. Did the review authors report any potential sources of conflict of interest, including any funding they received for conducting the review?	**Yes**	No	/	**Yes**	No	/	Yes	**No**	/	Yes	**No**	/	**Yes**	No	/	**Yes**	No	/
TOTAL SCORE (out of 16)	4	9		6	6		0	13		0	13		6	4		5	6	
Overall methodological quality (L = low, M = moderate, H = high)	Critically Low quality review			Critically Low quality review			Critically Low quality review			Critically Low quality review			Critically Low quality review			Critically Low quality review		
